# Directed -*in vitro*- evolution of Precambrian and extant Rubiscos

**DOI:** 10.1038/s41598-018-23869-3

**Published:** 2018-04-03

**Authors:** Bernardo J. Gomez-Fernandez, Eva Garcia-Ruiz, Javier Martin-Diaz, Patricia Gomez de Santos, Paloma Santos-Moriano, Francisco J. Plou, Antonio Ballesteros, Monica Garcia, Marisa Rodriguez, Valeria A. Risso, Jose M. Sanchez-Ruiz, Spencer M. Whitney, Miguel Alcalde

**Affiliations:** 1 Department of Biocatalysis, Institute of Catalysis, CSIC, Cantoblanco, 28049 Madrid, Spain; 2 División de Tecnología Química y Nuevas Energías, Centro del Tecnología Química, Repsol S.A, 28935 Móstoles, Spain; 3 Facultad de Ciencias, Departamento de Química Física, Universidad de Granada, 18071 Granada, Spain; 4 Research School of Biology, The Australian National University, Acton, Australian Capital Territory 2601 Australia

## Abstract

Rubisco is an ancient, catalytically conserved yet slow enzyme, which plays a central role in the biosphere’s carbon cycle. The design of Rubiscos to increase agricultural productivity has hitherto relied on the use of *in vivo* selection systems, precluding the exploration of biochemical traits that are not wired to cell survival. We present a directed -*in vitro*- evolution platform that extracts the enzyme from its biological context to provide a new avenue for Rubisco engineering. Precambrian and extant form II Rubiscos were subjected to an ensemble of directed evolution strategies aimed at improving thermostability. The most recent ancestor of proteobacteria -dating back 2.4 billion years- was uniquely tolerant to mutagenic loading. Adaptive evolution, focused evolution and genetic drift revealed a panel of thermostable mutants, some deviating from the characteristic trade-offs in CO_2_-fixing speed and specificity. Our findings provide a novel approach for identifying Rubisco variants with improved catalytic evolution potential.

## Introduction

Ribulose-1,5-bisphosphate carboxylase/oxygenase, Rubisco (EC 4.1.1.39), is the most abundant enzyme on earth, due to its necessity in plants, algae and wide distribution in bacteria^1^. In photosynthesis, the physiological role of this ancient enzyme (it dates back to the Precambrian era, around 3,500 million years ago) is the fixation of atmospheric CO_2_ into biomass through the Calvin cycle^2^. Specifically, Rubisco carboxylates the pentose sugar ribulose-1,5-bisphosphate (RuBP) and cleaves it into two molecules of 3-phosphoglycerate (3PGA), the central precursor for carbohydrate synthesis in plants^3^. Despite this important role in natural energy production, Rubisco is a slow biocatalyst with turnover rates as low as 1 to 10 s^−1^. The Rubisco carboxylation reaction is competitively inhibited by O_2_. The inability by Rubisco to fully discriminate between CO_2_ and O_2_ is due to their close electrostatic nature^4^ and the inability of Rubisco to directly bind either gas substrate in an enzyme Michaelis complex. The oxygenation reaction of RuBP produces 3PGA and 2-phosphoglycolate (2PG), the latter a compound that is inhibitory to other Calvin cycle enzymes^5^ necessitating its recycling back to 3PGA through energy demanding photorespiration^6,7^.

Given Rubisco’s catalytic inefficiency, the last decade has witnessed many attempts in laboratories worldwide to engineer more efficient Rubiscos in a drive towards more productive agriculture^8,9^. This challenge arises from the realisation that to meet the food demands of a growing global population we will have to produce more food in the next 50 years than has been consumed in the history of human kind^9,10^_._ The most promising results in Rubisco bioengineering have arrived through directed evolution, a biotechnological approach that emulates the process of natural evolution to tailor enzymes and metabolic pathways for industrial and environmental purposes^11,12^. To date, all the directed evolution studies reported on Rubisco have been assisted by *in vivo* -genetic- screening based on photosynthetic selection (*e*.*g*. *Rhodobacter capsulatus* and *Chlamydomonas reinhardtii*) and Rubisco dependent *Escherichia coli* (RDE) selection^6,13–15^. Using these strategies form I Rubisco (that comprise 8 large (RbcL) and 8 small (RbcS) subunits) and Rubisco forms II and III (each comprising between 2 and 10 RbcL-subunits) have been evolved to generate an array of variants, mostly with improved solubility^16–23^ and only more recently meaningful kinetic improvements^15,24^. These evolution campaigns have involved engineering cellular metabolism to be dependent on Rubisco activity for survival. In most instances this metabolic remodeling has reduced cell viability, lowered selection fidelity, constrained screening throughput and limited mutagenic screening to 25 °C^11,14,24^. Such factors have limited the suitability of these Rubisco evolution studies to improving thermal stability, a biophysical trait that is intrinsically connected to protein evolvability in terms of mutational tolerance, fitness and biological function^25^.

In this study, we report a robust directed -*in vitro*- evolution platform that allows Rubisco to be evolved independently of its host’s physiology. We reconstructed and resurrected several Rubisco nodes from the Precambrian era, dating from 3.2 to 1.9 billion years ago. The most recent common ancestor of proteobacteria was evolved, together with the modern form II Rubisco from *Rhodospirillum rubrum*, using a variety of evolution strategies aimed at improving thermostability that included adaptive evolution, focused evolution and neutral genetic drift. Rubisco mutant libraries were explored *in vitro* with a dual high-throughput screening (HTS) system that rapidly identified highly thermostable and functional variants. Comprehensive biochemical characterization of both the evolved extant and ancestral Rubisco mutants was undertaken to provide evidence for the first successful directed evolution of an ancestral -Precambrian- protein.

## Results

### Directed -*in vitro*- evolution of extant Rubisco

**Figure 1:**
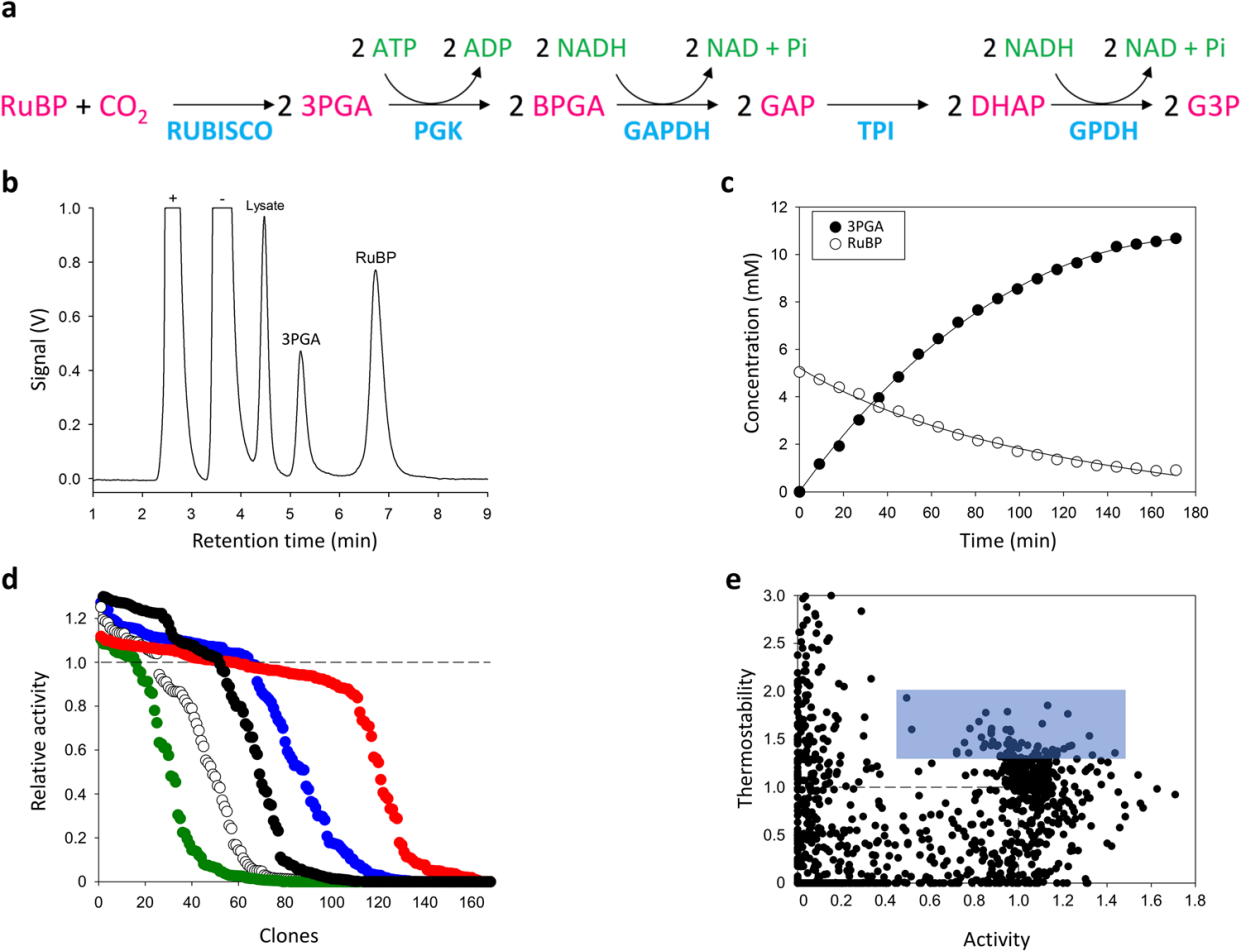
Dual HTS assay. (**a)** NADH-linked enzymatic assay of Rubisco activity. The reaction monitors the rate 3 PGA production from the carboxylation of Ribulose-1,5-bisphosphate (RuBP) by Rubisco. PGK, phosphoglycerokinase; BPGA, 1,3-bisphosphoglycerate; GAPDH, glyceraldehyde-3P dehydrogenase; GAP, glyceraldehyde-3P; TPI, triose-P isomerase; DHAP, dihydroxyacetone-P; GPDH, glycerol-3P dehydrogenase; G3P, glycerol-3P. (**b)** HPLC-ELSD chromatogram for the detection of 3PGA production/RuBP depletion by Rubisco (+and - represent cationic and anionic compounds in the sample, respectively) and (**c)**, the variation in the concentration of these two compounds during the reaction as measured by HPLC-ELSD. (**d)** Fitness landscapes for RubRr mutant libraries that are adjusted by varying the MnCl_2_ concentrations (in the case of Taq libraries) or by the amount of the DNA template (for mutazyme libraries). The relative RubRr activity of the clones is plotted in descending order and the dashed line shows the activity of the parental type in the assay: red circles, library I (mutazyme, 750 ng DNA template); blue circles, library II (Taq, 0.02 mM MnCl_2_); black circles, library III (mutazyme, 100 ng DNA template); white circles, library IV (Taq, 0.03 mM MnCl_2_); green circles, library V (Taq, 0.05 mM MnCl_2_). (**e)** Directed evolution landscape for thermostability and activity relative to the parental RubRr enzyme (dashed lines). The mutants selected for further re-screening are contained in the blue rectangle (using thresholds of 0.5- and 1.3-fold over RubRr activity and thermostability, respectively).

Form II Rubisco from the proteobacteria *Rhodospirillum rubrum* (RubRr) was chosen as the template for directed -*in vitro*- evolution and ancestral resurrection. RubRr comprises two 50 kDa RbcL subunits arranged into a functional RbcL_2_ dimer that does not require a Rubisco activase for metabolic repair. Accordingly, the folding and assembly of functional form II Rubisco are readily met in *E*. *coli*^26^, unlike the form I Rubisco isoforms of plants or algae. As RubRr production in *E*. *coli* can be toxic to growth^11^ the culture and induction conditions were optimized in a microtiter plate format for sufficient Rubisco expression without compromising host viability (*i*.*e*. ∼40 Units/g of lysate from clones grown in 96 well plates; One Unit is defined as the amount of Rubisco that catalyzes the transformation of 1μmol of RuBP per min). To evolve Rubisco *in vitro*, a dual HTS assay was established that couples both spectrophotometric and chromatographic detection under saturating CO_2_ conditions. The spectrophotometric method is a NADH-linked enzymatic depletion assay^27^ adapted to measure RuBP carboxylation activity in RubRr mutant libraries, (Fig. 1a). This assay was complemented with a new HPLC-ELSD (evaporative light scattering detection) ion pair method to accurately measure RuBP consumption and 3PGA formation by Rubisco (Fig. 1b,c). Both methods were validated by checking the responses and the coefficient of variation (<15%) in parental RubRr lysates from 96-well microculture plates (Supplementary Figs 1 and 2). Two consecutive re-screenings were employed to exclude false positives.

**Figure 2:**
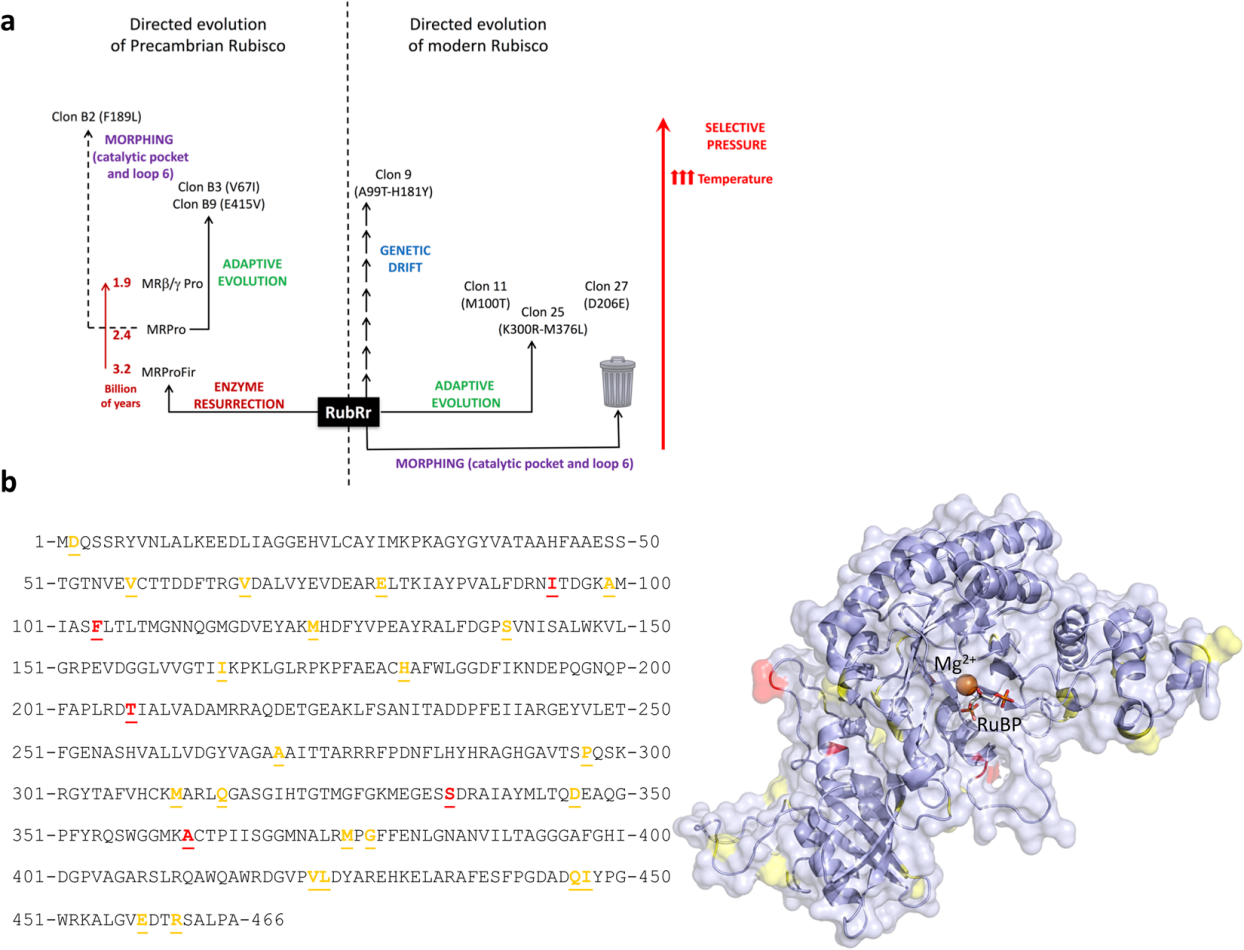
Directed -*in vitro*- evolution campaign. (**a)** General overview of the directed evolution campaigns carried out on Precambrian and modern (RubRr) form II Rubisco. (**b)** Neutral mutations identified in the amino acid sequence of RubRr (left) and mapped to its crystal structure (right). Mutations identified once are in yellow and those found in several clones are in red.

**Figure 3:**
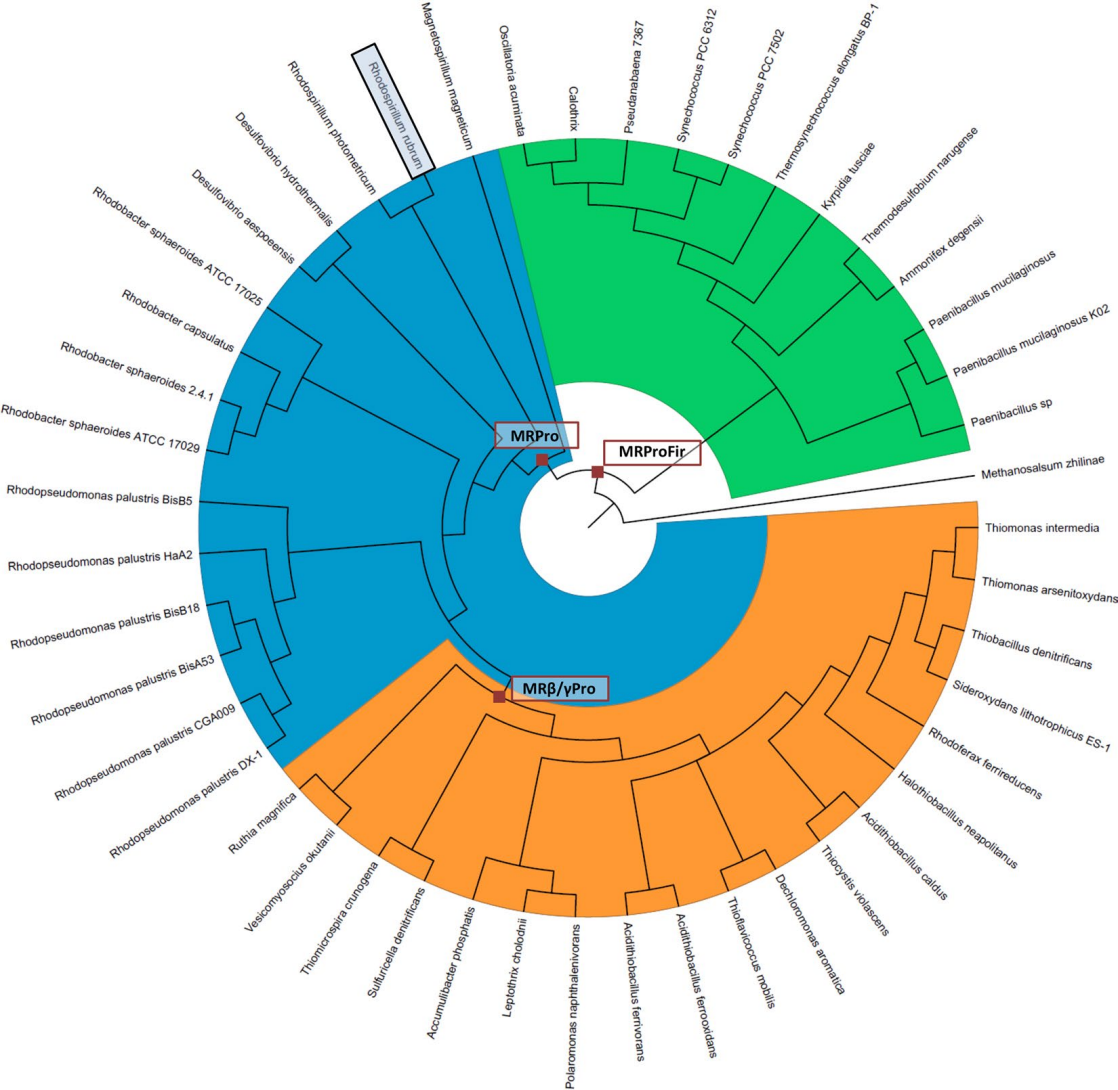
Phylogenetic tree generated from the amino acid sequences of 47 different form II Rubisco sequences. The colored areas represent the different bacterial phyla and classes as follows: Firmicutes (Green); α and δ proteobacteria (Blue); γ and β proteobacteria (orange). The brown squares highlight the nodes whose sequences were selected for resurrection and the reference *R*. *rubrum* Rubisco (RubRr) sequence is framed. See also Supplementary Figs 10–12.

**Figure 4:**
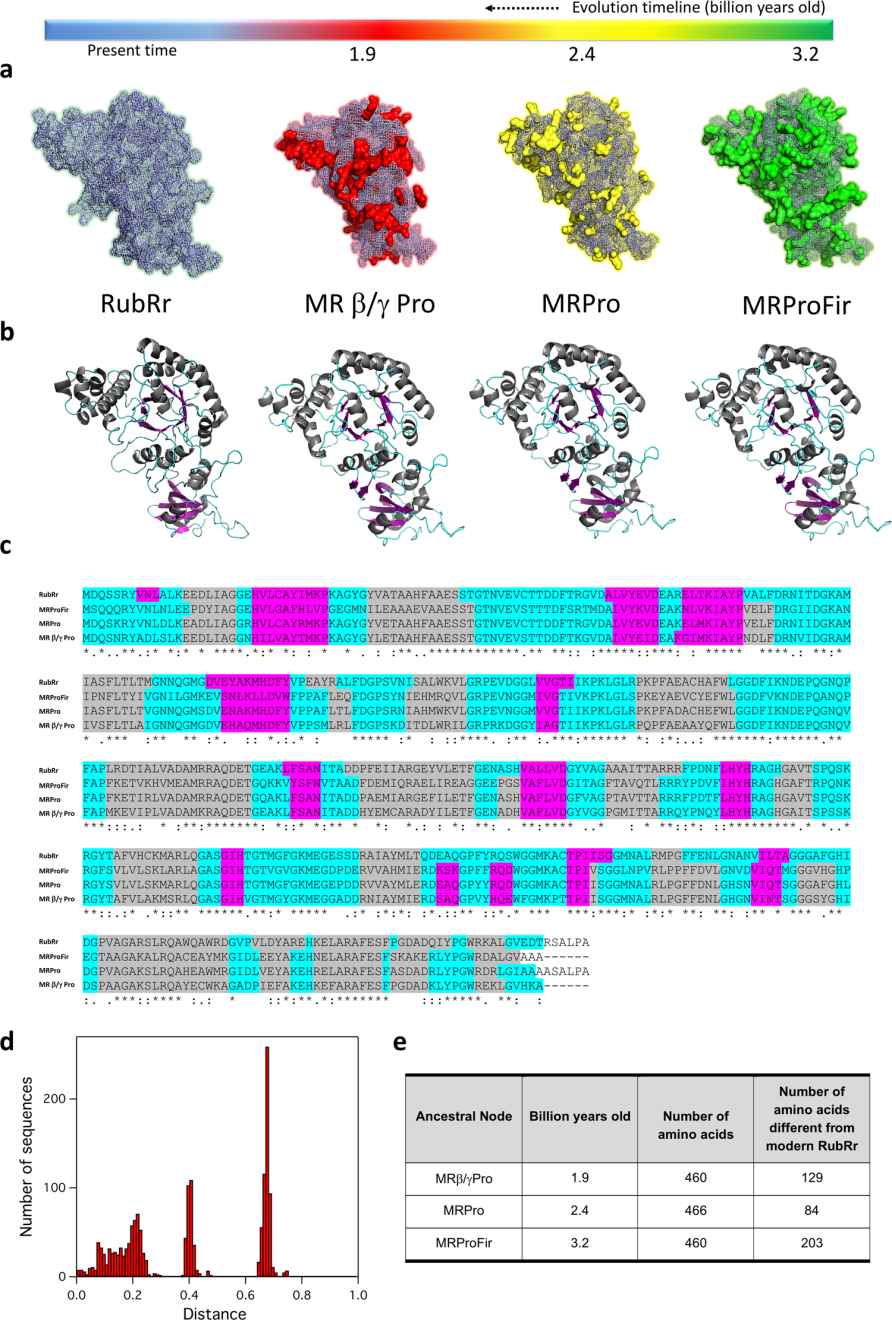
**C**omparison between modern RubRr and the ancestral -resurrected- Rubisco. (**a)** Amino acid differences (in surface mode) in MRProFir (green), MRPro (yellow) and MRβ/γPro (red) relative to RubRr. (**b)** 3D motifs in RubRr and ancestral Rubisco. (**c)** Multiple sequence alignment of RubRr and ancestral Rubiscos. Expected motifs are highlighted in colors. (pink, beta sheets; grey, alpha helices; blue, loops). The asterisk indicates positions with a fully conserved residue; colon indicates conservation between groups of strongly similar properties; period indicates conservation between groups of weakly similar properties. (**d**) Frequency distribution of the pairwise sequence distances for form II Rubisco sequences obtained from a database search using the RubRr as the query. The sequence distances are represented on the X axis and the number of sequences is reflected on the Y axis. (**e**) Number of amino acids different from RubRr and billion years old between the ancestral nodes. The multiple sequence alignment was performed with Clustal Omega V1.2.4 available at https://www.ebi.ac.uk/Tools/msa/clustalo/. The structures were modeled using the PDB code 9RUB and the Phyre2 server (Protein Homology/analogY Recognition Engine V 2.0: Kelley and Sternberg, 2009) available at www.sbg.bio.ic.ac.uk/phyre2.

This *in vitro* evolutionary platform was first tested by constructing a small set of RubRr mutant libraries with different mutational loads and biases (Fig. 1d). Depending on the mutagenic PCR conditions applied, mutational frequencies ranging from 12 to 73% of inactive clones were observed. 23 clones were selected randomly, and sequenced, to optimize the HTS assay conditions and Rubisco library mutation rate. To improve thermostability, the HTS assay and the microtiter expression system were used to evolve RubRr through different strategies: adaptive evolution, focused evolution, and neutral genetic drift (Fig. 2a). First, we determined the optimum temperature at which to stress the RubRr mutant libraries. The *T*_50_ (the temperature where the enzyme lost 50% of its initial activity after 10 minutes of incubation) was estimated to be 64 °C in the fresh lysates and this was the main selecting force used during screening (Fig. 1e). From this first adaptive evolution campaign, we identified three variants with improved stability (clone 11, clone 25 and clone 27), harboring the M100T, K300R-M376L and D206E mutations, respectively (Table 1; Fig. 2a; Supplementary Figs 3 and 4; Supplementary Table 1). It is worth noting that two of these four substitutions (M376L and D206E) were identified as consensus-ancestral mutations, as they were present in the ancestral nodes reconstructed from RubRr (see below: Figs 3 and 4), in close agreement with the stabilization observed when inserting back-to-ancestor mutations^28,29^.

**Table 1:** Biochemical characterization of extant and ancestral evolved Rubiscos.

Rubisco	*In vitro* Evolution strategy	*T*_m_ (°C)	[Rubisco] (% tsp^1^)	S_C/O (mol.mol_^−1^_)_	*K*_C_ (μM)	*k*_cat_^C^ (s^-1^)	*k*_cat_^C^/*K*_C_ (mM.s^-1^)	*K*_m_^RuBP^ (μM)
RubRr	Modern parent	70.7	28.1 ± 2.6	12.3 ± 0.2	105.2 ± 4.6	13.1 ± 1.1	125.2 ± 16	4.6 ± 1.5
Clone 9	Genetic drift	73.2	23.6 ± 1.3	11.6 ± 0.3	99.6 ± 1.1	12.9 ± 0.2	129.5 ± 2.9	5.4 ± 1.4
Clone 11	Adaptive evolution	74.5	31.0 ± 1.7	11.7 ± 0.2	318.7 ± 40.1	10.6 ± 0.9	33.6 ± 0.3	6.9 ± 0.9
Clone 25	Adaptive evolution	77.4	28.2 ± 1.2	12.7 ± 0.3	178.0 ± 4.1	8.1 ± 0.5	45.2 ± 1.9	45.9 ± 1.5
Clone 27	Adaptive evolution	74.2	34.6 ± 4.9	12.1 ± 0.2	111.5 ± 2.2	14.1 ± 1.1	126.5 ± 8.3	8.7 ± 0.8
MRPro	Ancestral parent	69.0	45.0 ± 4.7	6.5 ± 0.2	97.7 ± 3.2	4.0 ± 0.2	40.7 ± 1.1	158.3 ± 16.2
Clone B2	MORPHING	75.0	44.9 ± 6.9	6.9 ± 0.1	198.2 ± 15.4	1.4 ± 0.1	7.1 ± 0.7	375.1 ± 42.9
Clone B3	Adaptive evolution	70.0	41.4 ± 2.6	6.2 ± 0.1	105.1 ± 6.3	3.8 ± 0.5	36.3 ± 2.9	164.4 ± 13.8
Clone B9	Adaptive evolution	n.d.	56.5 ± 3.3	7.7 ± 0.6	94.9 ± 2.7	3.5 ± 0.1	36.6 ± 2.5	206.7 ± 14.8

^1^Tsp, total soluble protein.

We explored the versatility of the *in vitro* evolution platform to construct mutant libraries by neutral genetic drift, a valuable evolutionary strategy to enhance protein stability^30^. By accumulating neutral mutations that maintain wildtype function, functional polymorphic populations can be accrued while purging detrimental mutations, a process also known as purifying selection^31^. As a rule of thumb, we established a threshold of >75% of the wildtype activity in the mutational landscape. Accordingly, around 200 functional neutral clones were selected, pooled in each round of evolution, and subjected to further random mutagenesis. After screening over 6,000 clones from seven generations of neutral drift, we randomly picked 20 neutral variants from the last generation, which were sequenced and analyzed (Fig. 2b). On average, 1.65 neutral mutations were found in each clone, excluding silent mutations, consistent with the mutational windows described previously^32^. These mutations were mapped onto the crystal structure of RubRr and most found to locate on the enzyme surface of the protein, as expected, away from catalytic motifs (Fig. 2b). The exceptions included the mutation I165V within the catalytic pocket and the M311V, Q315H and S335G substitutions located in loop 6, a conserved flexible loop that closes over the catalytic site during catalysis. Among the neutral mutants analyzed, clone 9 (A99T-H181Y) showed improved thermodynamic stability (Table 1; Supplementary Fig. 3).

### Directed *in vitro* evolution of Precambrian Rubisco

The prediction of ancestral enzymes using phylogenetic algorithms is a plausible approach to recreate the metabolic phenotypes of primitive cells^33–35^. It is hypothesized that the courtship between ancestral enzyme resurrection and directed evolution could yield promising blueprints for adaptation to different environments and for new functions^36,37^. To test this, we undertook directed evolution of a predicted Precambrian Rubisco, one of the most ancient of enzymes known. The reconstruction of several form II Rubisco ancestral nodes was carried out based on the most recent common ancestor of β and γ proteobacteria (MRβ/γPro), the most recent common ancestor of all the proteobacteria (MRPro), and the most recent common ancestor of proteobacteria and the cyanobacteria firmicutes (MRProFir), which date back between 1.9 to 3.2 billion years (Figs 2a, 3 and 4). During the reconstruction process, the tree topologies for the two sequence groups that lie further from the query sequence (*i*.*e*. RubRr) were found to depart substantially from accepted phylogenies, in particular with respect to that reported in the TimeTree of Life^38^. Therefore, we used the TimeTree of Life to date the nodes in our phylogenetic tree. We hypothesized that the two groups most distant from the query sequence were the result of early horizontal transfer events and/or that they include other Rubisco isoforms. Consequently, we only retained the sequences belonging to the group closest to the query for sequence reconstruction. The three ancestral nodes were successfully resurrected (*i*.*e*. functionally expressed in *E*. *coli*) and the stronger activity of MRPro (2.4 billion years old) in preliminary biochemical measures of Rubisco activity in the cellular protein, made it the most promising candidate for directed evolution (Supplementary Fig. 5a).

To compare the *in vitro* evolution of ancestral and modern Rubisco, we constructed independent mutant libraries using MRPro and modern RubRr as templates. Identical mutational rates (1–4 mutations per kb) were applied to these libraries, as confirmed by DNA sequencing of a random sample of clones. The resulting evolution landscapes were quite different, producing 76% of inactive clones from RubRr and only 26% of unfolded variants for the ancestral MRPro enzyme (Supplementary Fig. 5b). This result highlights the marked tolerance of MRPro to the introduction of random mutations, supporting the hypothesized enhanced potential of evolving ancestral proteins towards beneficial phenotypes^37^. As a result, MRPro was included within the directed evolution program for improved stability, resulting in the isolation of the B3 clone coding a V67I substitution (Fig. 2a; Table 1; Supplementary Figs 3 and 6; Supplementary Table 1).

To compare the evolution potential of Precambrian and modern Rubisco, MRPro and RubRr were both subjected to structure-guided evolution by MORPHING, a focused random mutagenesis method developed for directed evolution in yeast and adapted here for *E*. *coli*^39^. By MORPHING, short structural motifs are targeted for random mutation without altering the remaining regions of the protein. It was reasoned that mutating residues integral to Rubisco catalysis may enhance stability, albeit likely at the cost of activity. MORPHING was therefore focused on modifying (i) the core catalytic pocket, a sequence block spanning 55 residues (K148-L204, according to RubRr numbering) that contains several amino acids directly involved in catalysis (*i*.*e*. K166, K168, K191, D193, E194); and ii) loop 6, a 12 amino acid segment (G323-D336) that shows different conformational states upon RuBP binding^40–42^ (Supplementary Fig. 7). This mutational strategy proved too aggressive for extant RubRr with no noticeable improved mutants and most fully inactive. In contrast functional MRPro mutants were obtained for both targeted regions, with clone B2 (coding a F189L substitution) comprising the most thermostable mutant (Fig. 2a; Table 1; Supplementary Figs 3 and 6; Supplementary Table 1).

### Biochemical characterization

**Figure 5:**
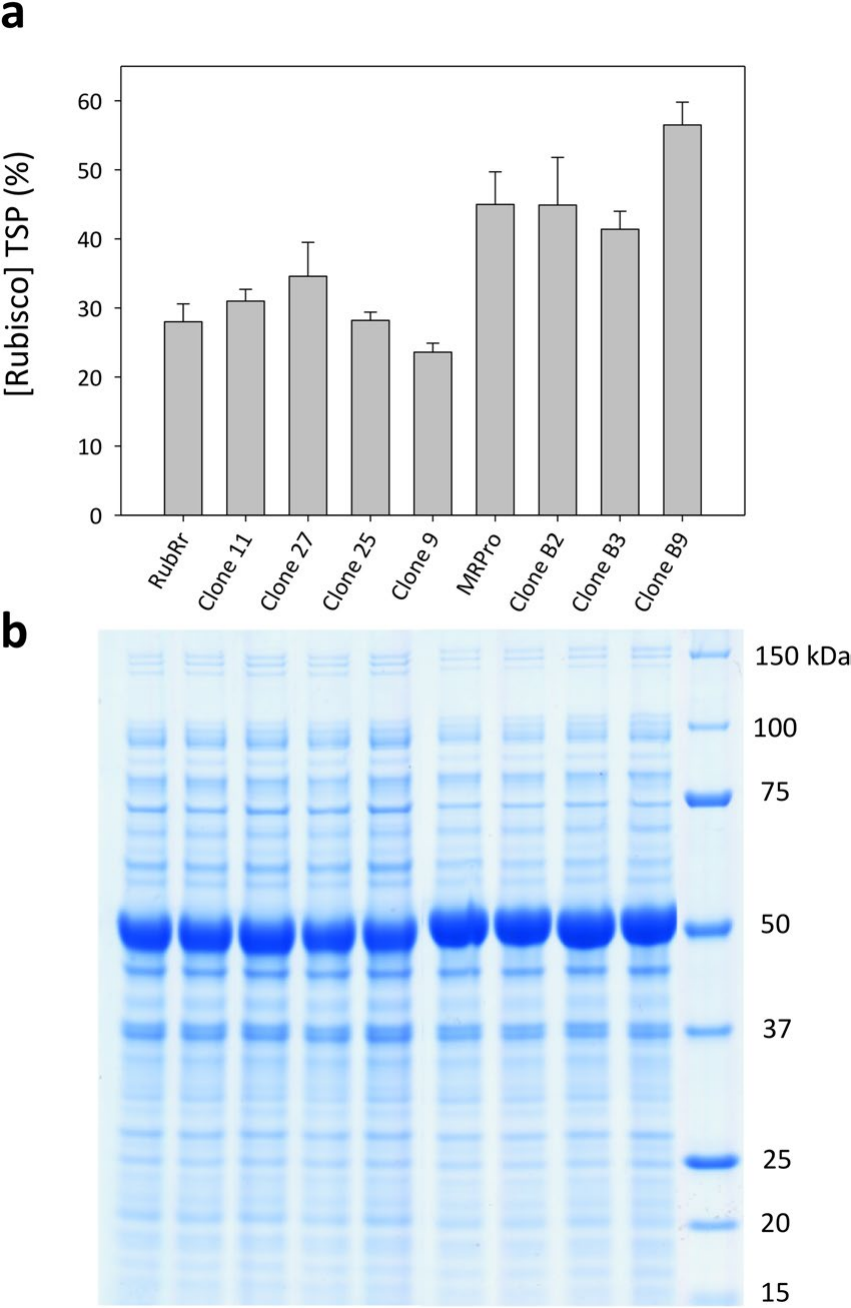
Expression of RubRr, ancestral Rubisco and their mutant offspring in *E*. *coli*. **(a)** Rubisco content as a percentage of the total soluble *E*. *coli* protein (TSP, %) determined by ^14^C-CABP binding and confirmed by (**b)** SDS-PAGE. Lane 1, RubRr; Lane 2, clone 11; Lane 3, clone 27; Lane 4, clone 25; Lane 5, clone 9; Lane 6, MRPro; Lane 7, clone B2; Lane 8, clone B3; Lane 9, clone B9; Lane 10, MW protein standard. The Rubisco RbcL subunit corresponds to the prominent 50 kDa band. Refer to Fig. 2a for the origins and mutations of each clone.

The ancestral MRPro node, the modern RubRr and their evolved variants were fully characterized in terms of their biogenesis (folding and assembly) in *E*. *coli*, thermostability and kinetic parameters. Functional RbcL_2_-Rubisco expression was quantified by stoichiometric binding of the ^14^C-CABP inhibitor to each catalytic site and confirmed by SDS-PAGE (Fig. 5**;** Table 1**;** Supplementary Fig. 8). Strikingly, the production of MRPro and its evolved mutants (clones B2, B3 and B9) in *E*. *coli* were greatly enhanced, comprising between 45% (in MRPro) to 56% (in clone B9) of the total soluble protein (tsp) as opposed to ∼30% for the modern RubRr enzyme (Table 1**;** Fig. 5). The improved assembly of Precambrian Rubiscos in a modern microorganism may be related to its predicted ancestral origin (2.4 Gyr ago). In this period primitive cells relied on a smaller set of enzymes with more promiscuous functions necessitating higher levels of expression to meet the metabolic requirements of the cell^36^.

Structural analyses revealed the stabilizing mutations were located on the surface of RubRr and quite distant from the conserved catalytic motifs, with exception of the F189L mutation in clone B2 (Supplementary Fig. 6**;** Supplementary Table 1). Detailed thermodynamic stability comparisons of the purified parental and select mutant enzymes (clones 9, 11, 25 and 27 from RubRr evolution; clones B2 and B3 from MRPro evolution, Fig. 2a) confirmed the improvement observed during the initial screening of the different *in vitro* evolution experiments. For example, the melting temperature (*T*_m_) of clone 25 derived by adaptive evolution outperformed that of RubRr by ∼7 °C (determined by DSC -differential scanning calorimetry) while the *T*_m_ of clones 11 and 27 showed improvements of ~4 °C (Table 1; Supplementary Fig. 3). Clone 9 from the neutral drift experiments showed a 2.5 °C enhancement in thermodynamic stability. Clone B2, the most thermostable variant derived from the focused evolution at the catalytic site of MRPro, showed a ∼6 °C increase in its *T*_m_.

A kinetic comparison of the parental and mutant RubRr and MRPro enzymes revealed the amino acid substitutions conferring thermodynamic stability adversely influence catalytic performance to varying extents (Table 1, Supplementary Fig. 9). Most notably, in comparison to RubRr, the ancestral MRPro and its evolved variants had ~2-fold lower specificities for CO_2_ over O_2_ (S_C/O_), more than 3-fold reductions in CO_2_-fixation rate (*k*_cat_^C^) and carboxylation efficiency (*k*_cat_^C^/*K*_C,_ where (*K*_C_) is the apparent *K*_m_ for CO_2_ in an O_2_–free atmosphere). This finding is in good agreement with the higher CO_2_ to O_2_ atmospheric ratio Precambrian Rubisco experienced prior to biogeochemical elevation of O_2_ in the atmosphere (estimated to occur 2.5 Gyr ago)^43^. Consistent with its focused evolution of catalytically critical domains, the MORPHING mutant Clone B2 showed >2-fold impediments in *k*_cat_^C^, *K*_C_ and affinity for substrate RuBP (*K*_m_^RuBP^) relative to MRPro (Table 1). This variation occurred with little effect (<6%) on S_C/O_ suggesting the catalytic chemistry of this resurrected mutant diverges significantly from modern Rubiscos where a canonical trade-off between the *k*_cat_^C^ and S_C/O_ is observed^3,44,45^.

Along these lines, the low carboxylation efficiency of our ancestral Rubiscos (from the Paleoproterozoic era, ∼2.4 Gyr ago) agrees well with the recent work reporting ancestral form I Rubiscos from the Mesoproterozoic (>1 Gyr ago)^35^, *viz*. 37.4, 28 and 40.7 mM^−1^ s^−1^ for the two ancestral nodes of form I Rubisco and MRPro, respectively. Accordingly, they can be considered as reliable proxies of true ancestral sequences.

## Discussion

Although Rubisco constitutes the primary bridge between the inorganic and organic phases of the biosphere’s carbon cycle, its carboxylation activity is inherently slow and flawed in its capacity to discriminate between CO_2_ and O_2_. These features often impair the rate of photosynthesis in plants making Rubisco a key target for engineered improvement as a means to increase agricultural productivity and carbon sequestration. The natural trade-off between the carboxylation activity and CO_2_/O_2_ specificity (S_C/O_) of Rubisco has hampered the selection of Rubisco mutants with the desired improvements in *k*_cat_^C^, *K*_C_ and S_C/O_. Paradoxically, despite its low carboxylation rates, modern Rubisco has a catalytic efficiency similar to the mean of all known enzymes^45^ but atypically necessitates large amounts of the enzyme (~25 to 50% of leaf protein) to sustain adequate rates of photosynthesis^3^. This provides evidence that the evolution of Rubisco kinetics may be naturally constrained thereby offering an explanation as to why attempts to evolve superior Rubiscos in the laboratory have met with limited success^15,24^ with most selecting for mutants with increased solubility^11^.

Yet there are reasons to be optimistic if we consider the favored catalytic behavior of Rubisco from some red algae that have the potential to improve plant photosynthesis and growth^26,46^. Moreover there is extensive catalytic diversity naturally found among, and within, the different Rubisco lineages despite all sharing a highly conserved catalytic site and reaction mechanism – suggesting kinetic improvement is not immutable. Certainly, we take hope from directed evolution studies using Rubisco-dependent *E*. *coli* (RDE) selection that have discovered unchartered domains in cyanobacterial RbcL_8_RbcS_8_ Rubisco and mutations in RbcL_10_ archaeal Rubisco that significantly improve their carboxylation properties^15,23,24^. However, the dependence on low temperature to slow cell growth of both RDE and photosynthetic screens used in directed evolution applications make them unsuitable to evolve the thermostability of Rubisco, a biophysical trait which may expand the sequence space available for improving catalytic function. Towards addressing this hypothesis, here we uniquely employed novel *in vitro* HTS assays to screen over 10,000 modern RubRr and resurrected Precambrian form II MRPro Rubisco clones in several campaigns of adaptive evolution, focused evolution and neutral genetic drift to identify a panel of thermostable variants. MRPro and its derivatives exhibited the properties consistent with an ancestral high CO_2_/low O_2_ environment (*i*.*e*. high solubility and impaired carboxylation properties) in addition to a higher mutational tolerance than RubRr. This suggests resurrected ancestral Rubiscos may serve as better substrates for evolutionary catalytic adaptation, possibly including variants harboring mutations in highly conserved catalytic regions as found here for the MORPHING mutant Clone B2. Notably this enhanced mutational robustness may arise independent of an improved stability as conformational flexibility may be a common feature of ancient proteins that are hypothesized to be more generalist in function^47^. Conversely, our study provides proof of concept into the versatility of using predicted Precambrian enzymes to address the promiscuity and evolvability for ancestral proteins using modern directed evolution techniques. While we postulate the approach of selecting form II Rubisco mutants suitable for evolving properties that improve catalytic function, the feasibility of translating these properties to other Rubiscos, in particular the form IB Rubisco in crops, remains uncertain. A promising feature of RubRr is that its catalytic mechanism and catalytic pocket amino acid composition is conserved in all Rubiscos. The minimal assembly requirements of RubRr that allow abundant expression in heterologous hosts, including in leaf chloroplasts, have also favored its use as a model for Rubisco structure-function studies and translational testing in plants. By contrast the assembly requirements of form IB plant Rubisco are not met by *E*. *coli*. This has necessitated the use of laborious plant genome transformation methods that restrict mutagenic study of plant Rubisco and preclude their suitability with high throughput directed evolution approaches^12^ Circumventing this obstacle now appears feasible with the demonstration that plant Rubisco assembly in *E*. *coli* requires the co-expression of multiple components from the assembly machinery from chloroplasts^48^, and possibly also its metabolic repair protein Rubisco activase if Rubisco activity is desired. As recently highlighted^49^, this breakthrough provides new opportunities for mutagenic study of plant Rubisco, including application of the directed *in vitro* evolution platform technology described in this study.

## Materials and Methods

### Alignment, phylogeny and ancestral sequence reconstruction

Bacterial Rubisco homologues were retrieved from the December 2013 release of the complete genomes database available at the NCBI (www.ncbi.nlm.nih.gov/genome). This search produced 295 protein sequences. We discarded sequences that were too long or too sort, resulting in a set of 201 sequences. The sequences were aligned using MUSCLE (available at https://www.ebi.ac.uk/Tools/msa/muscle/) and a distance matrix was then generated using one minus the sequence similarity as the parameter to assess the evolutionary distance between two sequences. The distribution for the calculated distances revealed three different groups and we found that several taxa were represented in the three groups of sequences, specifically: cyanobacteria; firmicutes; and α, β and γ proteobacteria. The sequences belonging to the group closest to the query were retained for sequence reconstruction resulting in 136 sequences. Since Mr. Bayes efficiency depends strongly on sequence set size, we selected 46 sequences for phylogenetic analysis. We made sure, however, that this subset provided a uniform coverage of the cyanobacteria, firmicutes, α-, β-, δ- and γ-proteobacteria taxa. A literature search confirmed that all the selected sequences belong to Rubisco type II. An Archean sequence was used as outgroup to generate a rooted tree making a total of 47 sequences (Supplementary Fig. 10). The tree topology and the branch lengths of the trees were estimated from these sequences using the Bayesian method implemented in version 3.1.2 of the program MRBAYES (available at http://mrbayes.sourceforge.net/). This analysis used the Jones substitution model and two independent Markov-chain Monte Carlo runs, each with four chains, and it was performed for 3,603,600 generations to ensure adequate convergence (0.037). The nodes obtained in the tree had posterior probabilities higher than 0.8. However, we targeted the three oldest nodes of the tree for sequence reconstruction, which had a probability of essential unity (Supplementary Fig. 11). The sequence reconstruction was performed using PAML version 4.4e. (available at http://abacus.gene.ucl.ac.uk/software/paml.html) and with the WAG evolutionary model (available at https://www.ebi.ac.uk/goldman-srv/WAG/). Subsequently, the proteins encoded by the most probable sequences at each of the three nodes targeted were prepared in the laboratory (*i*.*e*., “resurrected”) and characterized experimentally (Supplementary Fig. 12).

### Directed evolution

#### Library creation methods

A full description of library creation methods used in this study is provided in Supporting Information.

#### Preparing Mutant libraries

PCRs products were cleaned and concentrated. They were cloned behind the trc (trp-lac) promoter of the expression vector pTrcHis2 B using the Gibson Assembly Master Mix. *E*.*coli* XL1-Blue were transformed with the plasmid containing the RubRr, the MRPro or the corresponding mutant libraries, plated on LB-Amp agar and grown for 16 h at 37 °C. Individual colonies were picked into 44 mm deep well plates containing LB-Amp medium (0.1 mL). Each plate contained an internal standard with Rubisco parental type (column 7, rows A to H) and a negative control (lysate from *E*.*coli* transformed with pTrcHis2 B without Rubisco, column 6, rows A to D).

The plates (master plates) were incubated at 37 °C with shaking at 250 rpm and 80% relative humidity in a humidity shaker (Minitron-INFORS, Biogen, Spain). After 16 h, clones from this pre-culture were inoculated (using a cryo-replicator CR1000 from Enzyscreen, Haarlem, Netherlands) into deep well plates with fresh LB-Amp (lysate plates). After 5 hours of incubation at 37 °C, 250 rpm and 80% relative humidity, LB-Amp with IPTG was added (0.4 mL final volume). The plates were incubated at 25 °C with shaking at 250 rpm and 80% relative humidity for 16 h. The plates were centrifuged at 3000 *g*, the medium was discarded and the cell pellets were frozen at −80 °C. After 12 h the frozen cell pellets were re-suspended in a lysis mixture of Tricine-KOH buffer (0.4 mL, 100 mM, pH 8.0) containing lysozyme (0.25 mg/mL), DNase I (1.5 U/mL) and 10 mM MgCl_2_. After 60 min at 37 °C and 250 rpm the lysates were centrifuged at 3000 *g* and the supernatant was used for the dual HTS assay as well as for the thermostability screening protocol.

#### Dual HTS assay

60 µL of the supernatant from lysate plates were transferred with the help of a liquid handler station (Freedom EVO 100, TECAN, Männedorf, Schweiz) to the reaction plates. 50 µL of activation buffer (20 mM MgCl_2_ and 40 mM NaHCO_3_ in 100 mM Tris-KOH buffer pH 8.0) was added to each well of the reaction plate. Reaction plates were briefly stirred and after 15 min at room temperature 70 µL of the Rubisco reaction buffer was added. The reaction buffer contained 20 mM MgCl_2_, 40 mM NaHCO_3_, 0.5 mM RuBP (for spectrophotometric assay) or 3 mM RuBP (for HPLC-ELSD assay) in 100 mM Tris-KOH buffer, pH 8.0.

Spectrophotometric assay: The reaction plate was stirred briefly and after 4 min at room temperature 40 µL of each well were transferred to a new plate containing 40 µL of 80% (v/v) ethanol to quench the reaction. The plates were stirred and after 5 minutes 120 µL of the NADH depletion buffer was added. Spectrophotometric-depletion buffer composition: 1.5 mM MgCl_2_, 0.4 mM NADH, 0.5 mM ATP, 0.5 U/mL TPI, 0.5 U/mL αGPDH, 0.5 U/mL GAPDH and 5 U/mL PGK in 100 mM Tris-KOH buffer (pH 8.0). The depletion assay was followed in kinetic mode (340 nm) using a plate reader (SpectraMax Plus 384, Molecular Devices, Sunnyvale, CA) (Supplementary Figs 1 and 2).

HPLC-ELSD assay: Selected clones of the spectrophotometric assay were transferred from the lysate plate to a new reaction plate and analysed by the HPLC-ELSD (high-performance liquid chromatography with evaporative light scattering detector) assay. The reaction plate was stirred briefly and after 30 min at room temperature 400 mM acetic acid was added to stop the reaction. The HPLC conditions were 50% Buffer A (12 mM ammonium acetate, pH 6.0), 40% Buffer B (200 mM n-amylamine in buffer A, pH 6.0) and 10% C (acetonitrile). The final concentration of n-amylamine was 80 mM. The HPLC analysis was performed with a pump (Varian 9012, Canada) coupled to a Nucleosil C18 (250 × 4,6 mm, Sugelabor, Spain) kept at 30 °C. Autosampler (Hitachi L-2200, VWR, Spain) was set to 10 °C to prevent evaporation of the samples. The ELSD conditions were 118 °C and 3.2 L/min of nitrogen flow. Quantification was made with the aid of calibration curves of RuBP and 3PG. Chromatograms were analyzed with Varian Star v.6.41 software.

First re-screening: aliquots of 10 µL of the best clones were removed from master plates to inoculate in 90 µL of LB-Amp in new 96 deep well plates. Columns 1 and 12 (rows A and H) were not used. After 16 h of incubation at 37 °C and 250 rpm and 80% relative humidity, 10 µL were transferred to the adjacent wells, containing 90 µL of fresh LB-Amp and further incubated for 5 hours in the same conditions. Then 300 µL of LB-Amp with IPTG were added. The plates were incubated at 25 °C with shaking at 250 rpm and 80% relative humidity for 16 h. Accordingly, every single mutant was grown in 4 wells. Parent types were subjected to the same procedure (lane D, wells 7–11). Plates were assessed using the same protocol of the screening described above.

Second re-screening: An aliquot from the best clones of the first re-screening was inoculated in 10 mL of LB-Amp and incubated at 37 °C and 250 rpm for 16 h. Plasmids from these cultures were extracted with NucleoSpin Plasmid kit and they were transformed together with the parental type into *E*.*coli*. After 16 h at 37 °C, five colonies of every single mutant were picked, inoculated in 100 µL of LB-Amp and re-screened as described above.

### Screening assay for thermostability

RubRr and MRPro mutant libraries from adaptive evolution, focused evolution and a random sample of the outcome from the genetic drift campaign were subjected to a thermostability screening protocol. Lysate plates were duplicated with the help of the liquid handler station by transferring 85 µL of lysates to a thermocycler plate (Multiply PCR plate without skirt, neutral, Sarstedt, Germany) and 60 µL to the initial activity plate. Thermocycler plates were sealed with thermo resistant film (Deltalab, Spain) and incubated at 64 °C in the thermocycler (MyCycler, Bio-Rad Laboratories). Incubation took place for 10 min (so that the assessed activity was reduced ½ of the initial activity). Then, thermocycler plates were placed on ice for 10 min and further incubated for 5 min at room temperature. 60 µL of lysates were transferred from thermocycler plate to a new plate (residual activity plate) with the help of the robot. Each well of both residual and initial activity plates was filled with 50 µL of activation buffer (20 mM MgCl_2_ and 40 mM NaHCO_3_ in 100 mM Tris-KOH buffer pH 8.0) Plates were briefly stirred and after 15 min at room temperature 70 µL of the Rubisco reaction buffer was added and the plates subjected to the spectrophotometric assay as described above. Relative activities were calculated from the difference between the absorption after incubation and that of the initial measurements normalized against the parental type in the corresponding plate. Thermostability values came from the ratio between residual activities (RA) and initial activities (IA) values. Selected clones were subjected to HPLC-ELSD analysis and to two consecutive re-screenings as described above.

### Biochemical characterization

A full description of the biochemical characterization used in this study is provided in Supporting Information, including: Production and purification of variants, thermostability measurements, expression analysis, CO_2_ kinetics, RuBP kinetics, CO_2_/O_2_ specificity, DNA sequencing and protein modeling.

## Electronic supplementary material

Supporting Information
