# Supplementary material for: Directed -in vitro- evolution of Precambrian and extant Rubiscos
Source: Sci Rep. 2018 Apr 3;8:5532. doi: 10.1038/s41598-018-23869-3 (PMC5883036; doi:10.1038/s41598-018-23869-3)
Supplement: Supplementary file 1 — Supporting Information [file 41598_2018_23869_MOESM1_ESM.pdf]

Supporting information for:

Directed -*in vitro*- evolution of Precambrian and extant Rubiscos

Bernardo J. Gomez-Fernandez[1], Eva Garcia-Ruiz[1], Javier Martin-Diaz[1], Patricia Gomez de Santos[1], Paloma Santos-Moriano[1], Francisco J. Plou[1], Antonio Ballesteros[1], Monica Garcia[2], Marisa Rodriguez[2], Valeria A. Risso[3], Jose M. Sanchez-Ruiz[3], Spencer M. Whitney[4] and Miguel Alcalde[1*]

[1]Department of Biocatalysis, Institute of Catalysis, CSIC, Cantoblanco, 28049 Madrid, Spain.

[2]División de Tecnología Química y Nuevas Energías, Centro del Tecnología Química, Repsol S.A., 28935 Móstoles, Spain.

[3]Facultad de Ciencias, Departamento de Química Física, Universidad de Granada, 18071 Granada, Spain.

[4]Research School of Biology, The Australian National University, Acton, Australian Capital Territory 2601, Australia.

*Correspondence should be addressed to: Miguel Alcalde, Department of Biocatalysis, Institute of Catalysis, CSIC, Cantoblanco, 28049 Madrid, Spain. Phone: +34 915854806; Fax: +34 915854760; Email: malcalde@icp.csic.es

Table S1. Structural information of mutations introduced in extant and ancestral Rubiscos.

Rubisco	Mutation	Secondary structure motif	Relative position	Distance to catalytic pocket (Å)
Clone 9	A99T<br>H181Y	Loop (bridge)<br>α helix	Surface, N-terminal<br>Surface	20.5<br>10.2
Clone 11	M100T	Loop	N-terminal, interaction between both monomers	18.8
Clone 25	K300R<br>M376L	Loop<br>α helix	Surface, interaction of both monomers<br>Surface, in the access site of the substrate	26.9<br>16.6
Clone 27	D206E	α helix	Surface	13.9
Clone B2	F189L	β sheet	Catalytic pocket	4.5
Clone B3	V67I	Loop	Surface, N-terminal	40.4
Clone B9	E415V	α helix	Surface	20.8

SUPPLEMENTARY FIGURE LEGENDS

Figure S1. Depletion assay method. 1) Transfer and enzyme activation. The activation of Rubisco takes place upon the addition of the activation buffer. **2)** The activated Rubisco is mixed with the reaction buffer and the carboxylation of RuBP starts. **3)** 40 µL of the reaction plate is transferred to a new plate containing EtOH to stop the reaction. Thereafter, the NADH depletion buffer is added and the reaction is followed. Selected clones of the spectrophotometric assay are then transferred from the master plate to a new reaction plate and analysed by the HPLC-ELSD. Two consecutive re-screenings are performed (see further details in Materials and Methods Section).

Figure S2. Validation of the spectrophotometric assay. a) Coefficient of variance (CV) for the spectrophotometric assay. The landscape corresponds to 88 independent clones containing RubRr parental type, grown in microtiter format, lysated and measured with the NADH depletion assay. The activity of the clones is plotted in descending order, solid line indicate the average value of the whole plate and dashed lines indicate the CV of the assay. **b)** Screen-capture of the plate reader SpectraMax Plus 384 with the Software SoftMax Pro v5.2. The software is measuring the NADH depletion in 96 well plate RubRr mutant library, monitoring at 340 nm for 5 min. The values correspond to mili-Units per minute and are shown in negative mode due to the consumption of NADH. Highlighted are the internal standards used as controls in each plate: pink, RubRr parental type; blue, *Escherichia coli* lysates. The rest of the wells shows the diversity in the Rubisco mutant library.

Figure S3. Differential scanning calorimetric (DSC) unfolding curves. a) RubRr and MRPro. **b)** RubRr and clone 9 (from genetic drift campaign). **c)**

RubRr and clones 11, 25 and 27 (from adaptive evolution). **d)** MRPro and clones B2 and B3 (see Fig 2a for summary details and origins of each clone).

Figure S4. Molecular model of subunit RbcL1 (PDB code 9RUB) including the mutations introduced in modern RubRr. The Rubisco structure is shown as a pink cartoon while the RuBP substrate and the residues of the catalytic pocket are highlighted as orange and green sticks, respectively, and with Mg^{2+} as a red sphere. Numbering is relative to the RubRr sequence. The crystal structure of RubRr at a resolution 2.6 Å was used for modeling the mutants by Pymol (Schrodinger, LLC [http://www.pymol.org])

Figure S5. Activity of ancestral nodes and evolution landscapes. a) Activity of the ancestral Rubisco nodes relative to MRPro (in %) measured in a microplate (grey) and flask (black) format. **b)** Evolution landscapes for RubRr (red circles) and MRPro (green circles) adaptive mutant libraries. The activity of the clones is plotted in descending order and the dashed line shows that of the parental type.

Figure S6. Molecular model of subunit RbcL1 including the mutations introduced in ancestral MRPro. The Rubisco structure is shown as a light brown cartoon while the RuBP substrate and the residues of the catalytic pocket are highlighted as orange and green sticks, respectively, and with Mg^{2+} as a red sphere. Highlighted are the positions of mutations F189L (clone B2), V67I (clone B3) and E415V (clone B9). The model was made using the Phyre2 server (Protein Homology/analogY Recognition Engine V 2.0: Kelley and Sternberg, 2009) available at www.sbg.bio.ic.ac.uk/phyre2. The outcome model was used for modeling the mutants by Pymol (Schrodinger, LLC [http://www.pymol.org]).

Figure S7. MORPHING in RubRr. a) Surface of the subunit RbcL1 with the regions selected for MORPHING highlighted in blue and the non-mutated areas in pink: left, catalytic pocket region; right, loop 6 region. **b)** Detail of the secondary motifs that form the catalytic pocket (Left) and loop 6 (Right). Some of the conserved residues involved in catalysis (catalytic pocket) and in the closure over the substrate (loop6) are labeled, and highlighted as sticks.). The model was made using the PDB code 9RUB

Figure S8. SDS PAGE analysis of Rubisco production in *E. coli.* a) Total cellular and **b)** soluble cellular protein separated by SDS-PAGE highlighting the expression and solubility of RubRr, MRPro and their derived mutants in *E. coli.* Lane 1, RubRr; Lane 2, clone 11; Lane 3, clone 27; Lane 4, clone 25; Lane 5, clone 9; Lane 6, MRPro; Lane 7, clone B2; Lane 8, clone B3; Lane 9, clone B9; Lane 10, MW protein standard. The Rubisco RbcL subunit corresponds to the 50kDa band. Refer to Fig 2a for the origins and mutations of each clone.

Figure S9. Measuring the CO_2-fixation rate and K_m for CO_2. Representative plots comparing the variation in the response of $^{14}CO_2$-fixation rate to differing [CO_2] for RubRr and MRPro under anoxic conditions at 25°C. The data from three independent experiments for each enzyme were fitted to the Michaelis-Menten equation (indicated by the lines shown) to derive the apparent K_m for CO_2 (K_C) and the maximal rate of carboxylation (V_C^{max}). The Rubisco-catalytic site content in each experiment was quantified by [^{14}C]-2-CABP use to normalise the extrapolated maximal carboxylase activities (k_{cat}^C) measured in each experiment. Data are used to derive the parameters shown in Table 1.

Figure S10. Rubisco sequences used for ancestral reconstruction. GI numbers were accessed from GenBank.

Figure S11. Inferenced evolutionary tree with the posterior probability at each node.

Figure S12. Robustness of the reconstructed ancestors as measure of the distribution of posterior probability.

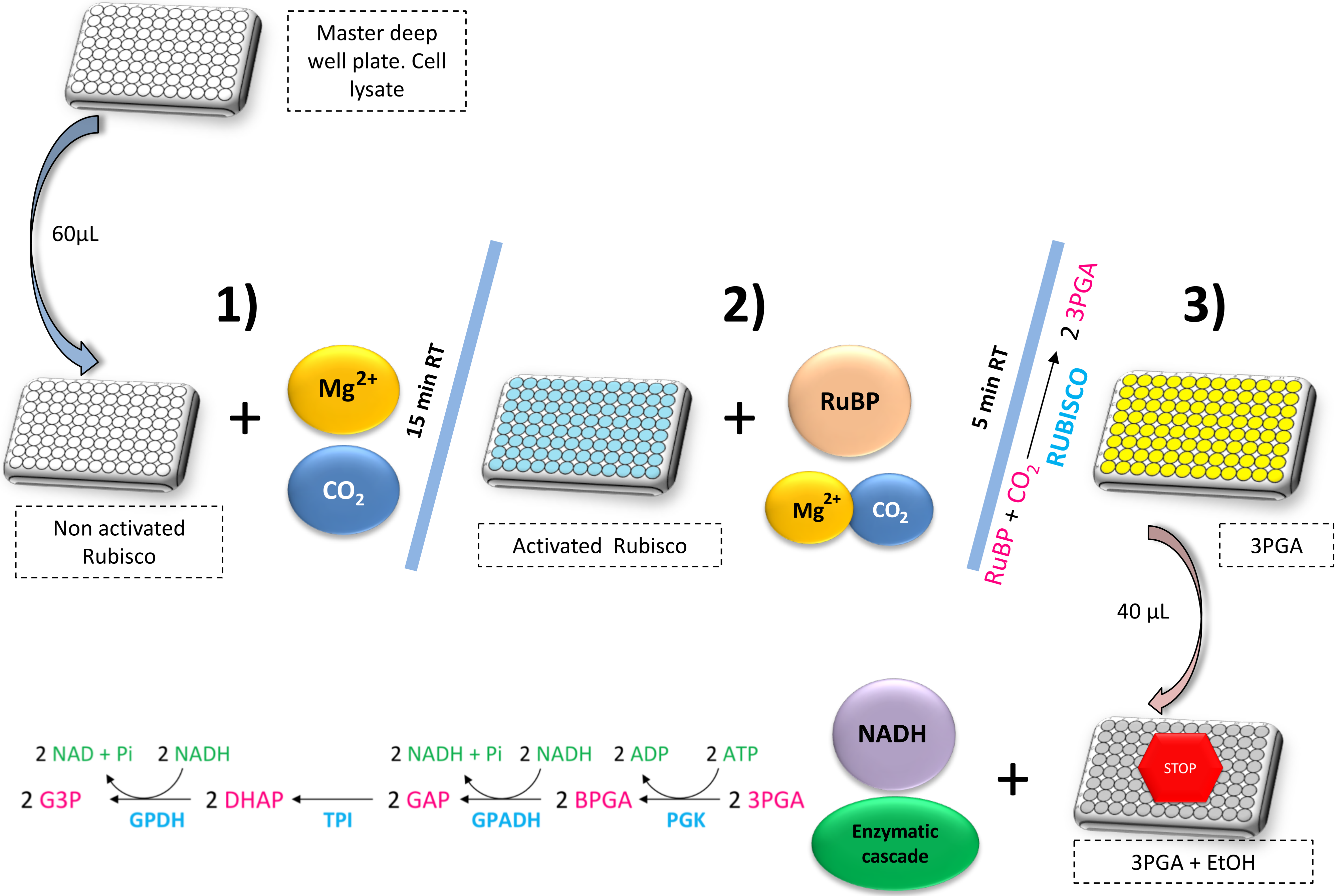
Master deep well plate. Cell lysate
60µL
1)
Non activated Rubisco
Mg2+
CO2
15 min RT
Activated Rubisco
2)
RuBP
Mg2+
CO2
5 min RT
RuBP + CO2
RUBISCO
2 3PGA
3)
3PGA
40 µL
STOP
3PGA + EtOH
NADH
Enzymatic cascade
2 NAD + Pi
2 NADH
2 G3P
GPDH
2 DHAP
TPI
2 NADH + Pi
2 NADH
2 GAP
GPADH
2 ADP
2 ATP
2 BPGA
PGK
2 3PGA

Fig. S2

a

b

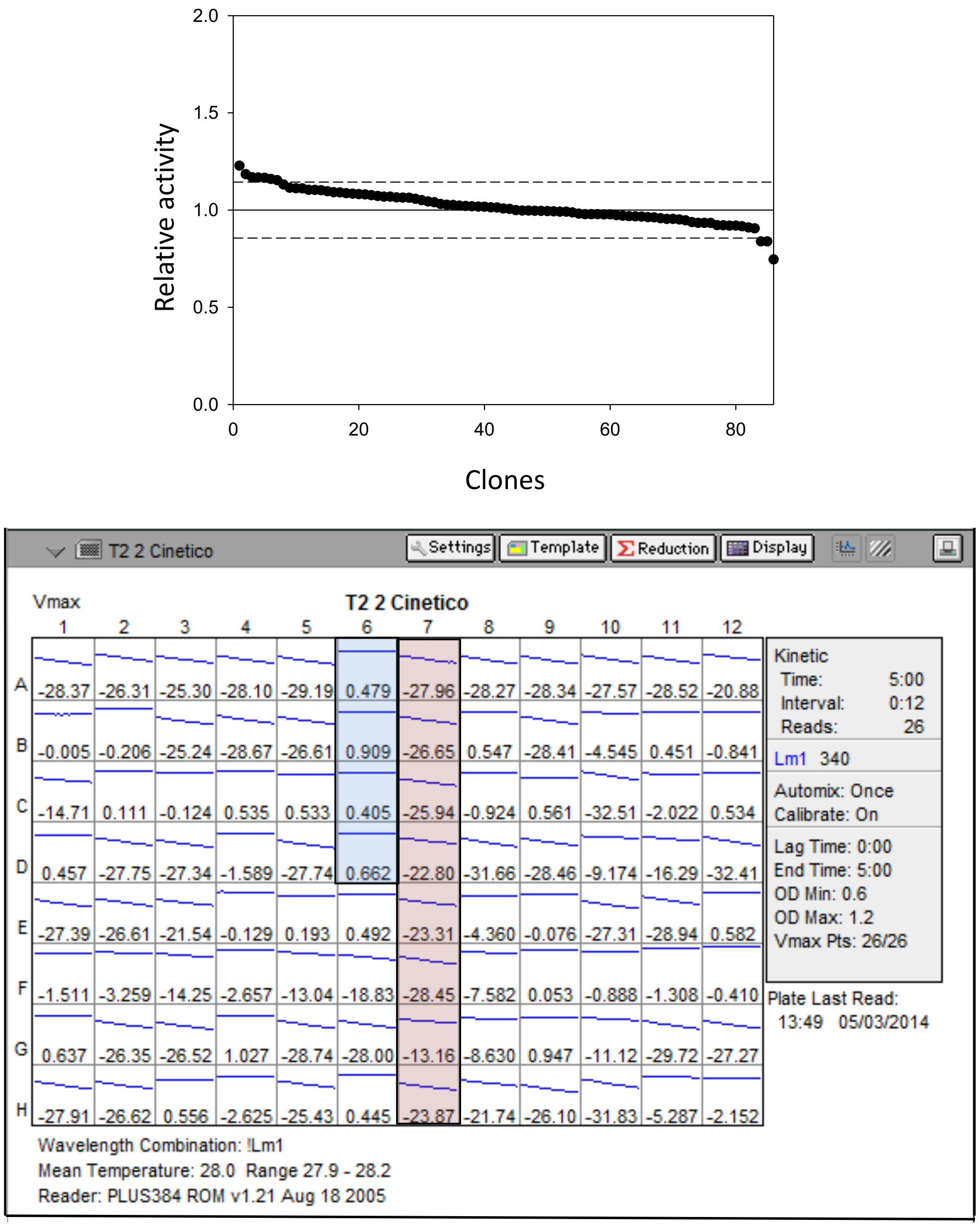
2.0
1.5
1.0
0.5
0.0
Relative activity
0
20
40
60
80
Clones
T2 2 Cinetico
Settings
Template
Reduction
Display
Vmax
T2 2 Cinetico
1 2 3 4 5 6 7 8 9 10 11 12
A -28.37 -26.31 -25.30 -28.10 -29.19 0.479 -27.96 -28.27 -28.34 -27.57 -28.52 -20.88
B -0.005 -0.206 -25.24 -28.67 -26.61 0.909 -26.65 0.547 -28.41 -4.545 0.451 -0.841
C -14.71 0.111 -0.124 0.535 0.533 0.405 -25.94 -0.924 0.561 -32.51 -2.022 0.534
D 0.457 -27.75 -27.34 -1.589 -27.74 0.662 -22.80 -31.66 -28.46 -9.174 -16.29 -32.41
E -27.39 -26.61 -21.54 -0.129 0.193 0.492 -23.31 -4.360 -0.076 -27.31 -28.94 0.582
F -1.511 -3.259 -14.25 -2.657 -13.04 -18.83 -28.45 -7.582 0.053 -0.888 -1.308 -0.410
G 0.637 -26.35 -26.52 1.027 -28.74 -28.00 -13.16 -8.630 0.947 -11.12 -29.72 -27.27
H -27.91 -26.62 0.556 -2.625 -25.43 0.445 -23.87 -21.74 -26.10 -31.83 -5.287 -2.152
Kinetic
Time: 5:00
Interval: 0:12
Reads: 26
Lm1 340
Automix: Once
Calibrate: On
Lag Time: 0:00
End Time: 5:00
OD Min: 0.6
OD Max: 1.2
Vmax Pts: 26/26
Plate Last Read:
13:49 05/03/2014
Wavelength Combination: !Lm1
Mean Temperature: 28.0 Range 27.9 - 28.2
Reader: PLUS384 ROM v1.21 Aug 18 2005

Fig. S3

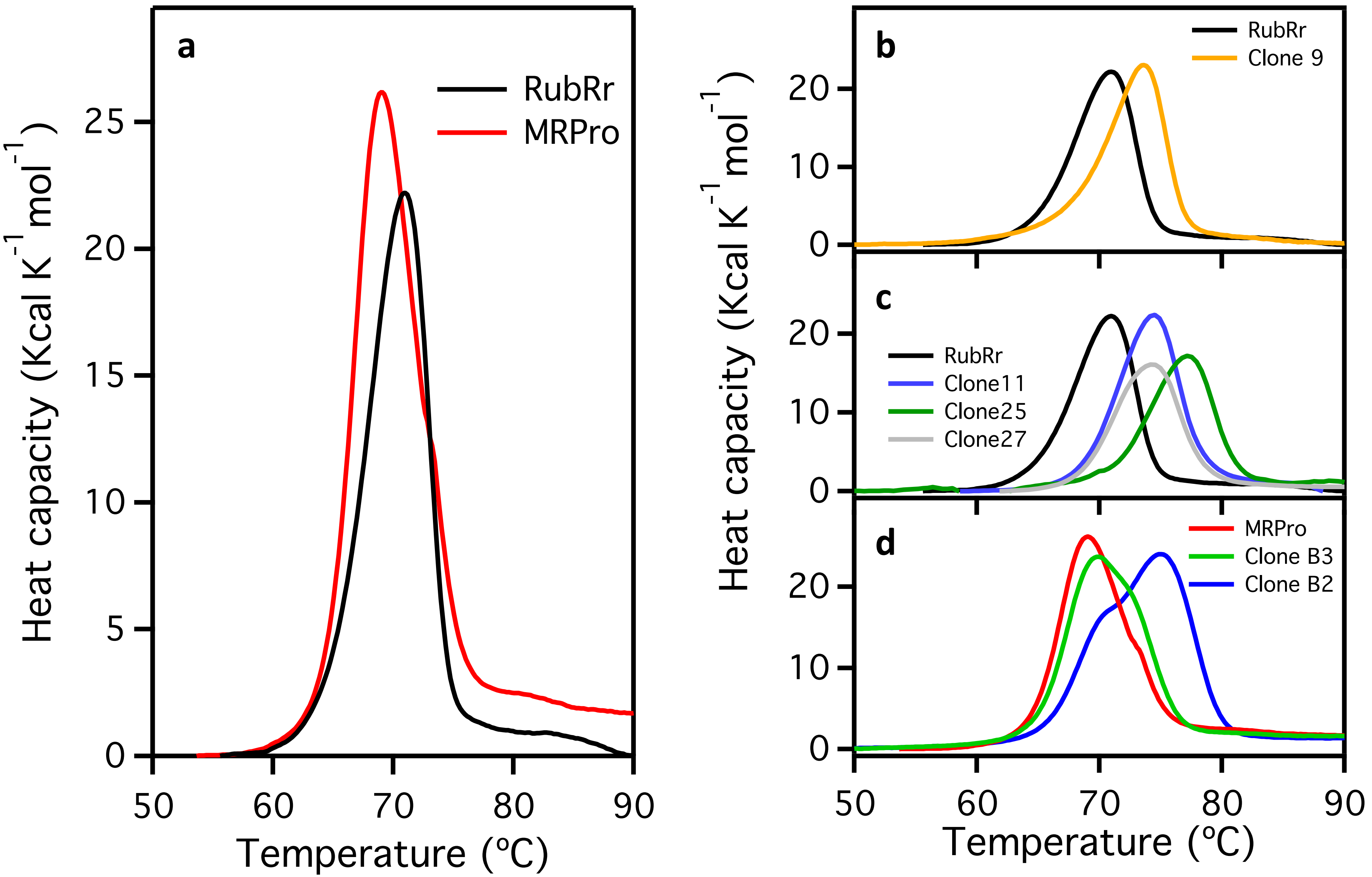

a
RubRr
MRPro
Heat capacity (Kcal K^{-1} mol^{-1})
0
5
10
15
20
25
50
60
70
80
90
Temperature (°C)
b
RubRr
Clone 9
c
RubRr
Clone11
Clone25
Clone27
d
MRPro
Clone B3
Clone B2

Fig. S4

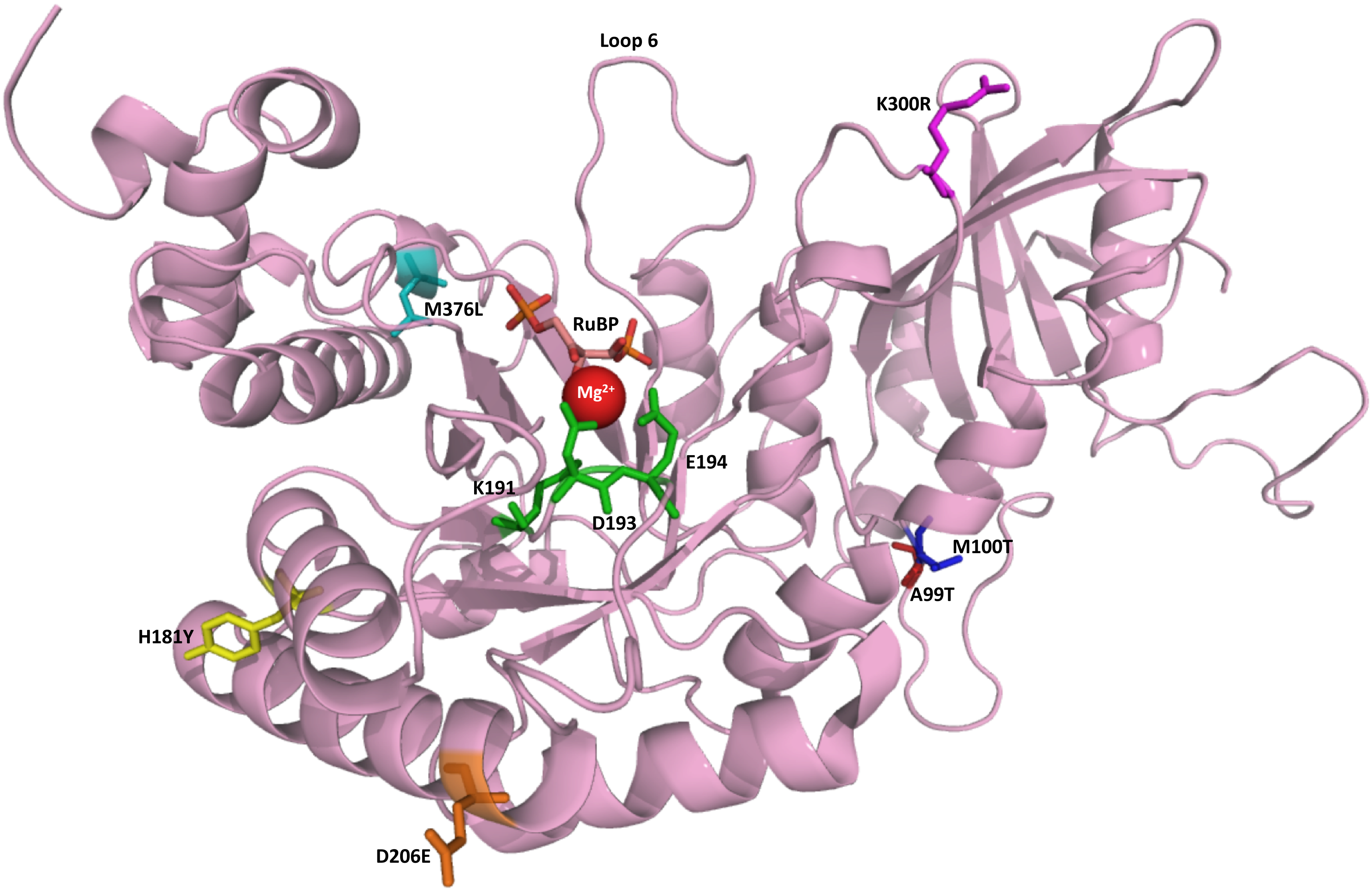
Loop 6
K300R
M376L
RuBP
Mg2+
E194
K191
D193
M100T
A99T
H181Y
D206E

Fig. S5

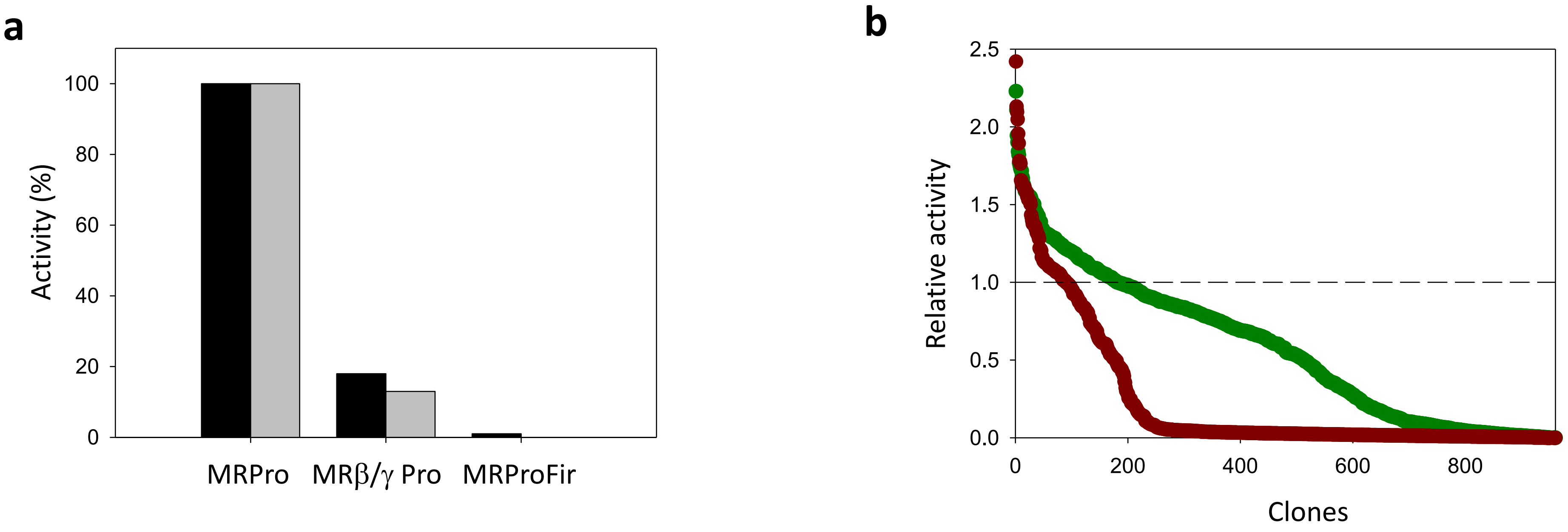

a
Activity (%)
100
80
60
40
20
0
MRPro
MRβ/γ Pro
MRProFir
b
Relative activity
2.5
2.0
1.5
1.0
0.5
0.0
0
200
400
600
800
Clones

Fig. S6

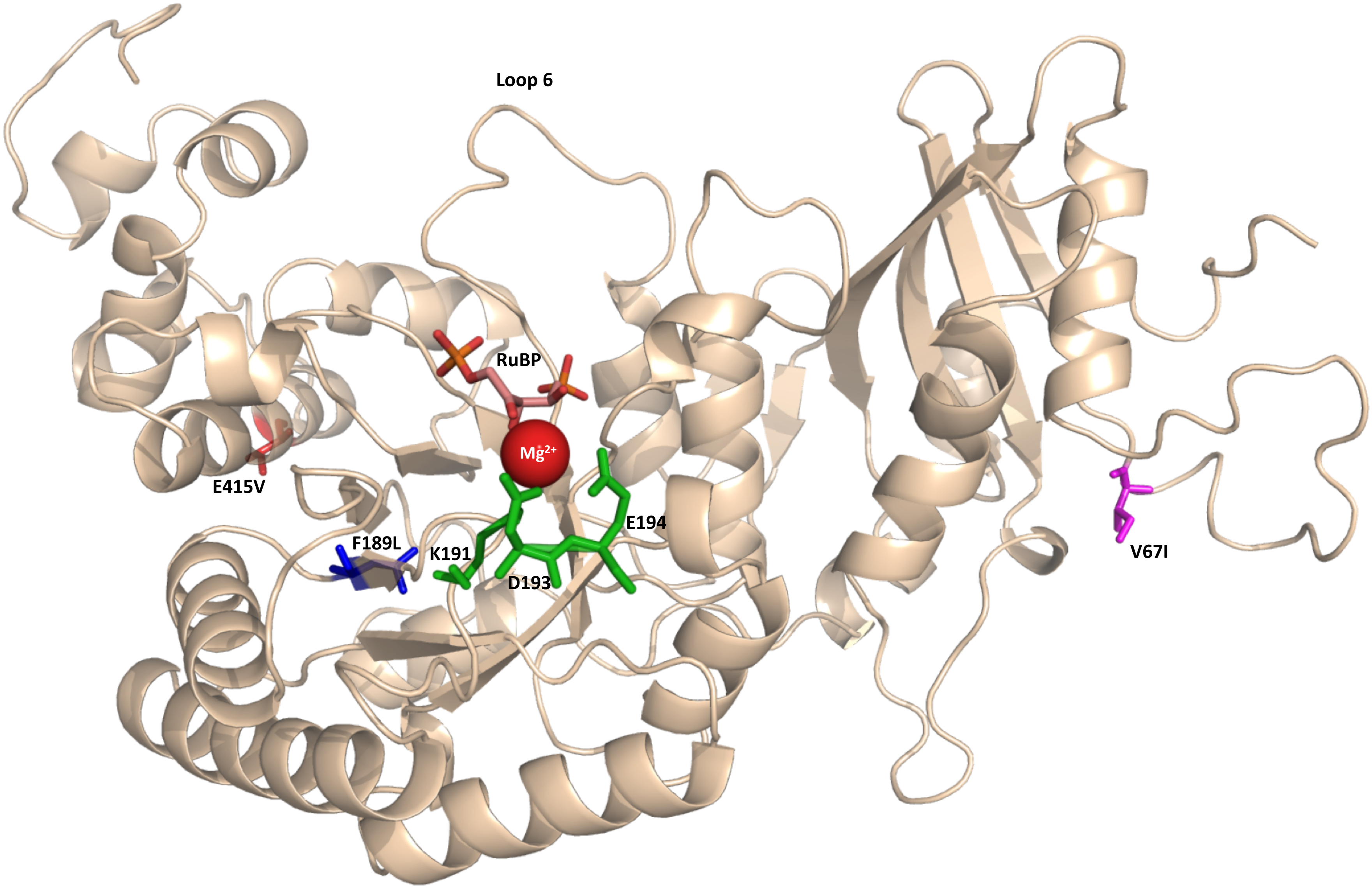

Fig. S7

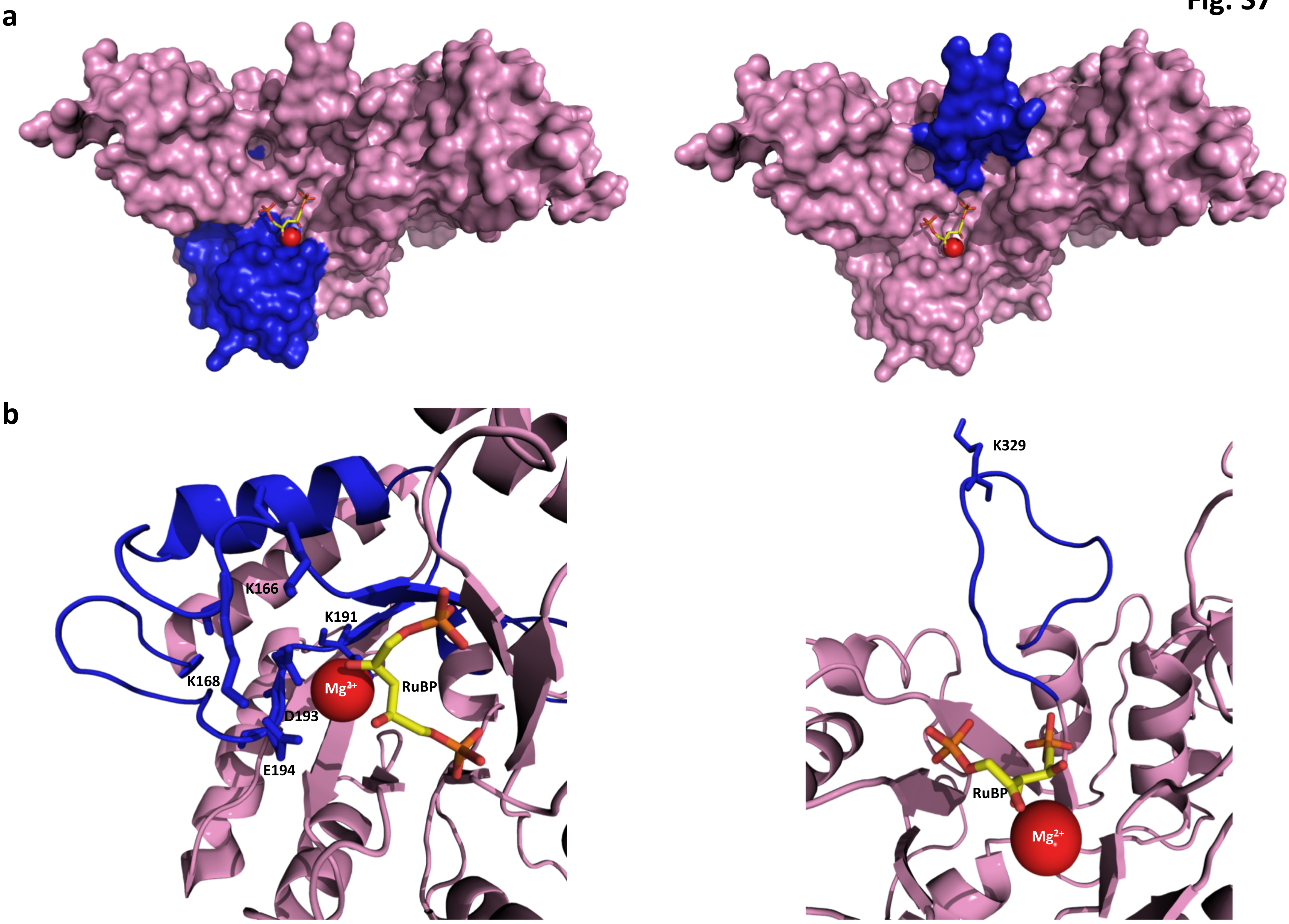

Fig. S8

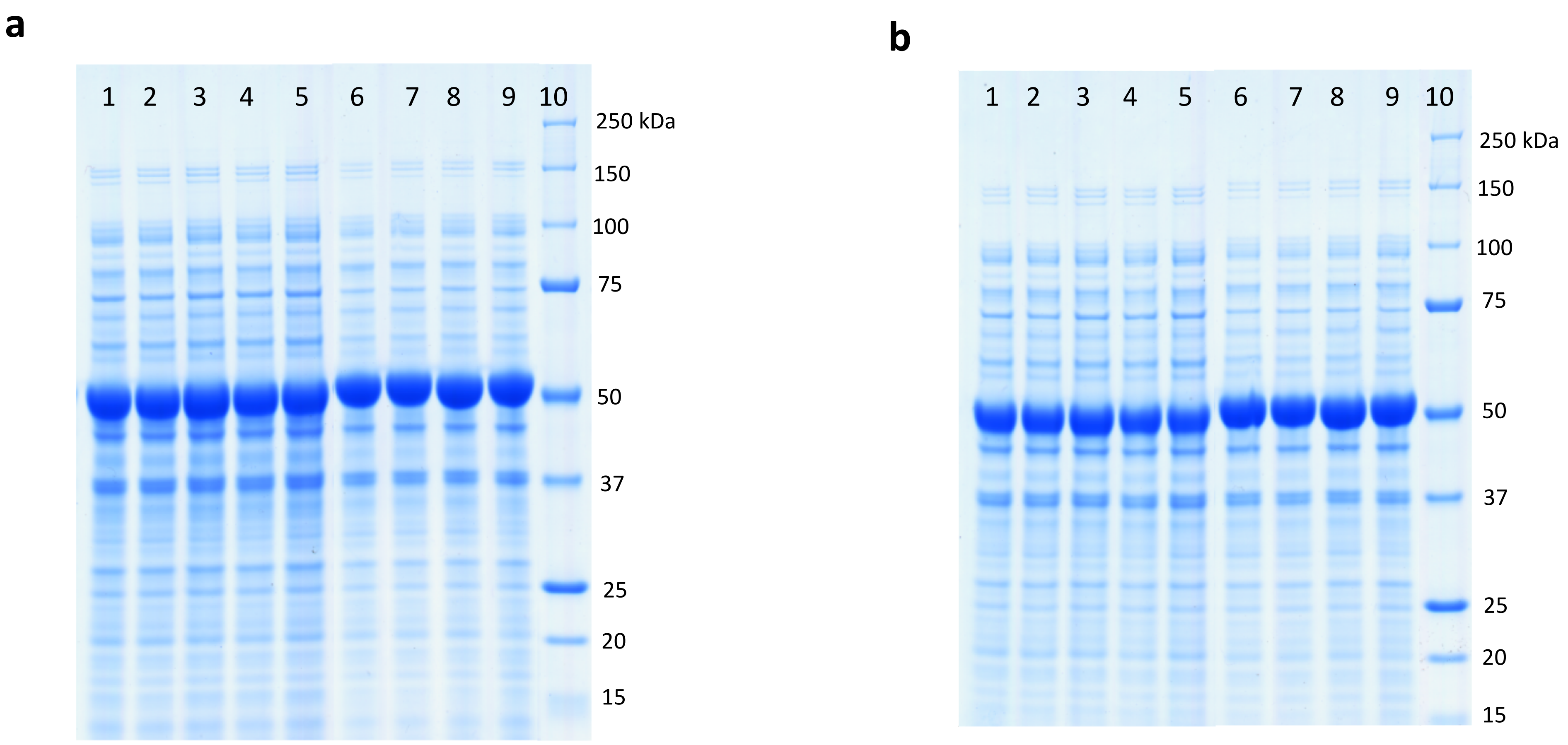
a
1 2 3 4 5 6 7 8 9 10
250 kDa
150
100
75
50
37
25
20
15
b
1 2 3 4 5 6 7 8 9 10
250 kDa
150
100
75
50
37
25
20
15

Fig. S9

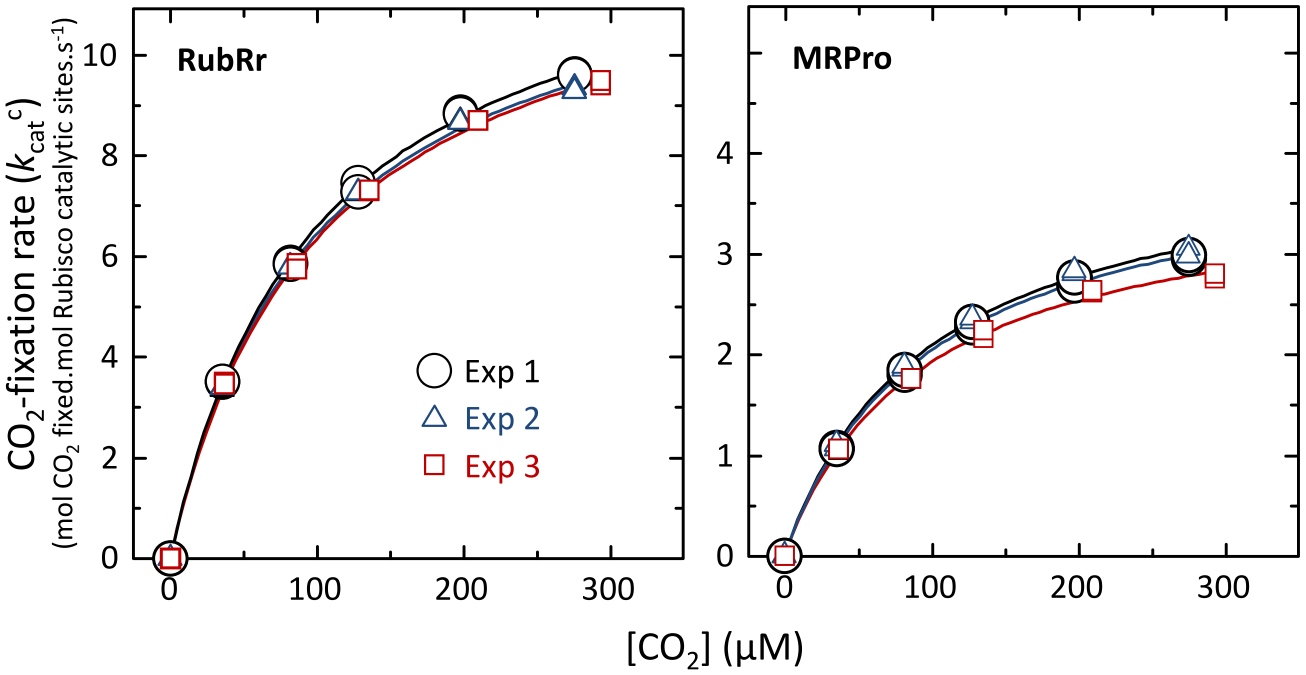
RubRr
MRPro
Exp 1
Exp 2
Exp 3
CO_2-fixation rate (k_{cat}^{c})
(mol CO_2 fixed.mol Rubisco catalytic sites.s^{-1})
[CO_2] (μM)
0
2
4
6
8
10
1
3
100
200
300

Fig. S10

GI number
332295273
260893699
295697414
428217181
428211462
428299658
427713058
428221690
22299049
261406831
337749006
386724715
317154242
436842937
89900184
296136113
410693894
74318656
291612494
261855695
340782403
390952417
71909249
431932595
198283921
344199398
121604879
171060437
257092457
543964646
78484769
336476213
148244796
118602694
316936114
39937699
115524692
90424392
86748076
91975534
294677366
126464741
77465277
146280021
384263572
83593735
83311795

Fig. S11

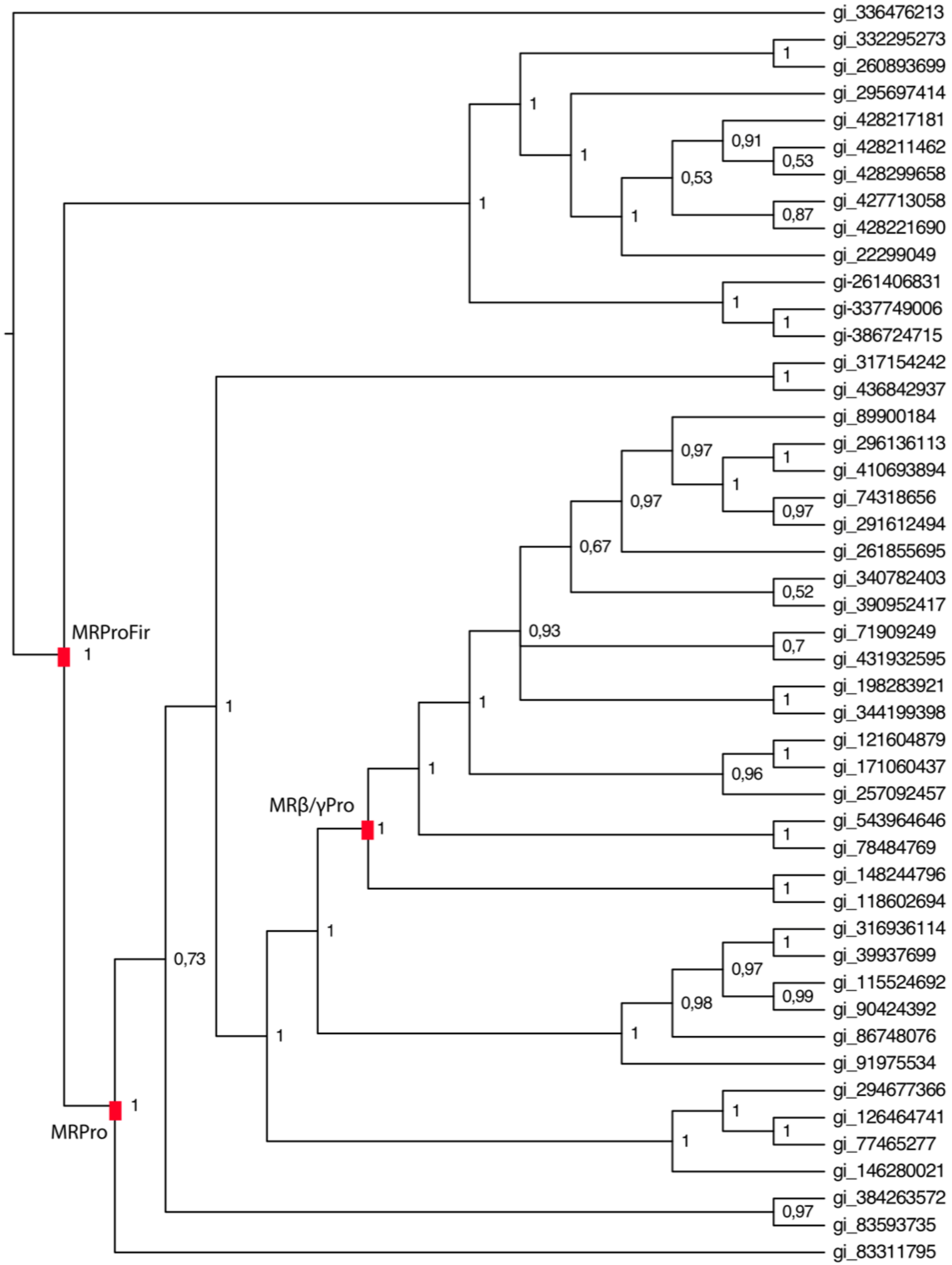
gi_336476213
gi_332295273
gi_260893699
gi_295697414
gi_428217181
gi_428211462
gi_428299658
gi_427713058
gi_428221690
gi_22299049
gi-261406831
gi-337749006
gi-386724715
gi_317154242
gi_436842937
gi_89900184
gi_296136113
gi_410693894
gi_74318656
gi_291612494
gi_261855695
gi_340782403
gi_390952417
gi_71909249
gi_431932595
gi_198283921
gi_344199398
gi_121604879
gi_171060437
gi_257092457
gi_543964646
gi_78484769
gi_148244796
gi_118602694
gi_316936114
gi_39937699
gi_115524692
gi_90424392
gi_86748076
gi_91975534
gi_294677366
gi_126464741
gi_77465277
gi_146280021
gi_384263572
gi_83593735
gi_83311795
MRProFir
MRβ/γPro
MRPro
0,91
0,53
0,53
0,87
0,97
0,97
0,97
0,67
0,52
0,93
0,7
0,96
0,97
0,99
0,98
0,73
0,97

Fig. S12

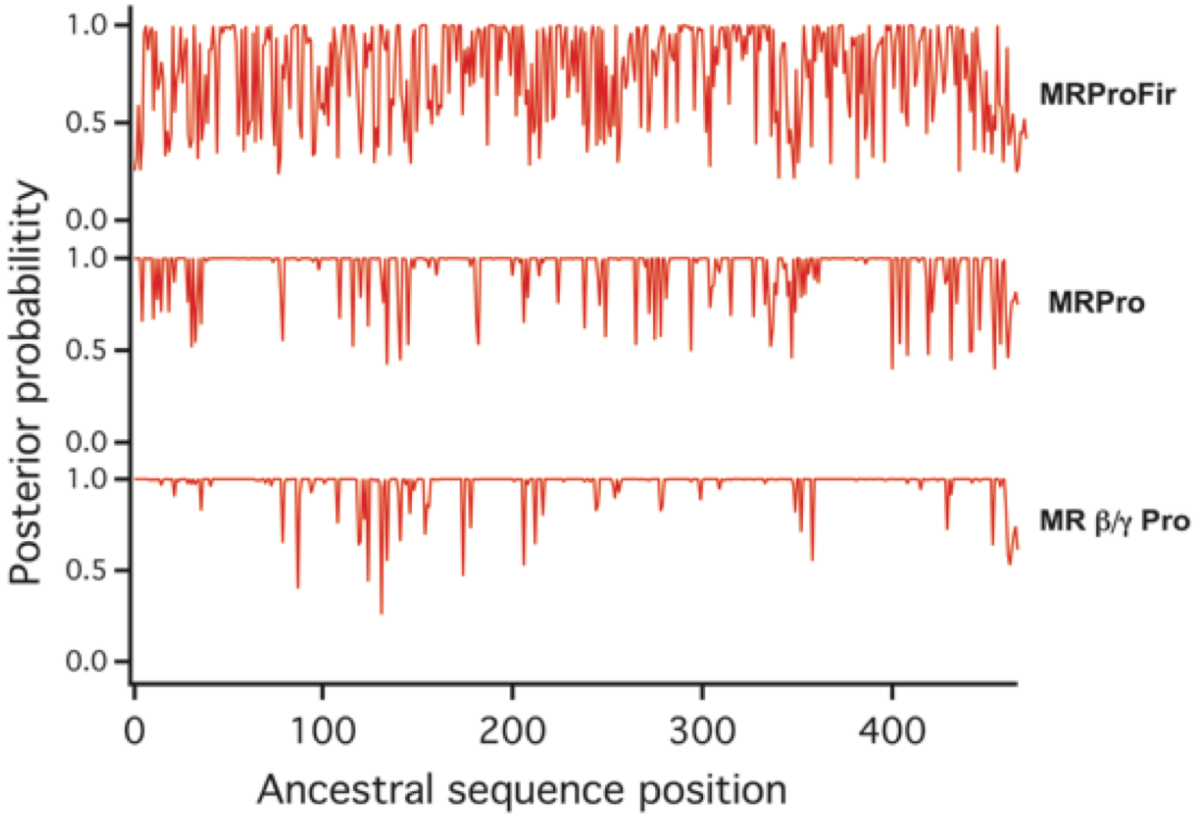
Posterior probability
Ancestral sequence position
1.0
0.5
0.0
0
100
200
300
400
MRProFir
MRPro
MR β/γ Pro

SUPPORTING MATERIAL AND METHODS

Genes, enzymes and reagents. RubRr (GeneBank: X00286.1), MRProFir, MRPro and MRβ/γPro were synthesized by ATG biosynthetics (Merzhausen, Germany). Taq polymerase, protease inhibitor mixture for bacterial cells, 3-phosphoglyceric acid (3PG), NADH, ATP and enzymes PGK, GAPDH, TPI, GPDH were purchased from Sigma-Aldrich (St. Louis, USA). N-amylamine and 2-phosphoglyceric acid were from Acros Organics (Thermo Fischer, NJ, USA) whereas ribulose-P_2 (RuBP) was purchased from Santa Cruz Biotechnology (Santa Cruz, USA). The *E. coli* XL1-Blue competent cells, the GeneMorph II Kit (mutazyme II polymerase) and the Pfu DNA Polymerase were from Agilent (Santa Clara, CA, USA). NucleoSpin Plasmid kit was purchased from Macherey-Nagel (Düren, Germany). The Zymoclean Gel DNA Recovery Kit was obtained from Zymo Research (Orange, CA, USA). The restriction enzymes NcoI-HF and Sal I, the DNAse I and the Gibson Assembly Master Mix were purchased from New England Biolabs (Hertfordshire, UK). Lysozyme was purchased from Amresco (Solon, USA). The IPTG was purchased from Thermo Fisher (Vantaa, Finland). The Gelcode Blue reagent, dye binding Pierce Coomassie Plus kit and NuPAGE Bis-Tris Gels were purchased from Invitrogen Thermo Fisher, MA USA. The unstained protein standard and the low melting point preparative agarose were purchased from Bio-Rad, CA, USA. RuBP unlabeled and [1-^{3}H]RuBP-labeled, were synthesized and purified as described (Kane et al., 1998). [^{14}C]2-Carboxyarabinitol-1,5-biphosphate ([2-^{14}C]CABP) was synthesized as described (47). The scintillant was from UltimaGold, Packard Bioscience, CT, USA. All chemicals were of reagent-grade purity all other reagents were of the highest purity grade.

Culture media. Luria-Bertani (LB) medium was prepared with 1% (w/v) peptone, 0.5% (w/v) yeast extract, 1% (w/v) NaCl and 100 μg/mL ampicillin.

Microplate expression medium contained 0.3 mM Isopropyl β-D-1-thiogalactopyranoside (IPTG) in LB. Flask expression medium contained 1 mM IPTG in LB.

Library creation methods: General aspects. For each generation PCR fragments were cleaned, concentrated and loaded onto a low melting point preparative agarose gel (Bio-Rad, Hercules, CA), and then purified using the Zymoclean gel DNA recovery kit (Zymo Research, Orange, CA). PCR products were cloned into the plasmid pTrcHis2 B from Invitrogen (California, USA) fused to a 6xHis-tag at the C-terminus using the Gibson Assembly Master Mix. pTrcHis2 B was linearized at the corresponding restriction sites with NcoI-HF and Sal I, then cleaned and purified as described for PCR products.

Error prone PCR (*ep*PCR) for adaptive evolution, focused evolution and neutral genetic drift was carried out using a gradient thermocycler (Mycycler, Biorad, USA). The cycling parameters unless others specified were: 94°C for 2 min (1 cycle), 94°C for 0.45 min, 50°C for 0.30 min, 72°C for 1.30 min (28 cycles) and 72°C for 10 min (1 cycle). The cycling parameters for high fidelity PCR in focused evolution were: 94°C for 2 min (1 cycle), 94°C for 0.30 min, 55°C for 0.30 min, 72°C for 1.30 min (28 cycles) and 72°C for 10 min (1 cycle). The primers used for amplification unless others specified were RubDir sense (5' TAAATAAGGAGGAATAAACCATGGACC 3') and RubRev antisense (5' TGATGATGATGATGATGGTCCGCCG 3') for RubRr; 62Dir sense (5' CGATTAAATAAGGAGGAATAAACCATGGATCAGTCTAAACGTTACG 3´) and 62Rev antisense (5' CTCAATGATGATGATGATGATGGGCAGGCAGGGCGCTTGCC 3') for MRPro.

*ep*PCR with Taq/$MnCl_2$: To find an appropriate mutational rate, several mutant libraries were prepared at different concentrations of $MnCl_2$ in a 50 μL

final volume containing: pTrcHis2 B-RubRr 2,82 ng/µL, 90 nM RubDir, 90 nM RubRev, 0.3 mM dNTPs (0.075 mM each), 3% (v/v) dimethylsulfoxide (DMSO), 1.5 mM $MgCl_2$, increasing concentrations of $MnCl_2$ (0.01, 0.02, 0.03 and 0.05 mM) and 0.05 U/µL Taq polymerase. For adaptive evolution, focused evolution and genetic drift, independent mutant libraries of RubRr and MRPro were prepared in a 50 µL final volume with pTrcHis2 B-RubRr 0.055 ng/µL or pTrcHis2 B-MRPro 0.055ng/µL, 90 nM of each primer, 0.3 mM dNTPs (0.075 mM each), 3% (v/v) dimethylsulfoxide (DMSO), 1.5 mM $MgCl_2$, 0.03 mM $MnCl_2$ and 0.05 U/µL Taq polymerase.

*ep*PCR with GeneMorphII: To find an appropriate mutational rate, several mutant libraries were prepared with different concentrations of pTrcHis2 B-RubRr (50, 100, 200 and 750 ng), 37 nM RubDir, 37 nM RubRev, 0.8 mM dNTPs (0.2 mM each), 3% (v/v) dimethylsulfoxide (DMSO), and 0.05 U/µL Mutazyme II.

Focused evolution by MORPHING: The MORPHING method (36) was adapted to *E. coli* with several modifications. Four different PCRs were performed for each library, two high fidelity PCRs upstream and downstream the mutagenic block, one mutagenic PCR (loop 6 or catalytic pocket) and one assembly PCR. The primers used for the upstream high fidelity PCR were RubDir sense and RubCatRev2 (5' GCACTTTCCACAGGGCCGAGATATTG 3') or RubL6Rev2 antisense (5' GGTATAGCCGCGCTTGGACTGG 3') for MORPHING in the catalytic pocket or the loop 6 of RubRr, respectively; 62dir sense and 62CatRev2 (5' CTTTCCACATATGGGCAATATTGC 3') or 62L6Rev2 antisense (5' AGAGTAGCCGCGCTTCGACTGG 3') for MORPHING in the catalytic pocket or the loop 6 of MRPro, respectively. The primers used for the downstream high fidelity PCR were RubRev antisense and RubCatDir2 (5' CTTGCGCGACACCATCGCCCT 3') or RubL6Dir2 sense (5'

AGGCCCAGGGGCCGTTCTACC 3') for MORPHING in the catalytic pocket or the loop 6 of RubRr, respectively; 62rev antisense and 62CatDir2 (5' GACCATCCGATTAGTCGCTGATGCG 3') or 62L6Dir2 sense (5' GATGAGGCACAGGGCCCCTAT 3') for MORPHING in the catalytic pocket or the loop 6 of MRPro, respectively. Upstream and downstream high fidelity PCR reactions contained 0.25 μM of each primer, 1 mM dNTPs (0.25 mM each), 3% (v/v) dimethylsulfoxide (DMSO), 0.2 ng/μL of pTrcHis2 B-RubRr or pTrcHis2 B-MRPro template and 0.05 U/μL Pfu polymerase. The primers used for the mutagenic PCR were: RubCatDir1 (5' CAATATCTCGGCCCTGTGGAAAGTGC 3') or RubL6Dir1 sense (5' CCAGTCCAAGCGCGGCTATACC 3') and RubCatRev1 (5' AGGGCGATGGTGTCGCGCAAG 3') or RubL6Rev1 antisense (5' GGTAGAACGGCCCCTGGGCCT 3') for MORPHING in the catalytic pocket or the loop 6 of RubRr, respectively; 62CatDir1 (5' GCAATATTGCCCATATGTGGAAAG 3') or 62L6Dir1 sense (5' CCAGTCGAAGCGCGGCTACTCT 3') and 62CatRev1 (5' CGCATCAGCGACTAATCGGATGGTC 3') or 62L6Rev1 antisense (5' ATAGGGGCCCTGTGCCTCATC 3') for MORPHING in the catalytic pocket or the loop 6 of MRPro, respectively. Mutagenic PCR reactions contained 0.09 μM of each primer, 0.3 mM dNTPs (0.075 mM each), 0.37 ng/μL of pTrcHis2 B-RubRr or pTrcHis2 B-MRPro template, 3% (v/v) dimethylsulfoxide (DMSO), 1.5 mM $MgCl_2$, 0.1 mM $MnCl_2$ and 0.05 U/μL Taq polymerase. The assembly PCR was done in two PCR-steps. The cycling parameters for the first part of the assembly PCR were 98°C for 2 min (1 cycle), 98°C for 0.30 min, 45°C for 0.30 min, and 72°C for 2 min (15 cycles). This PCR reaction contained 0.25 μM of each primer, 1 mM dNTPs (0.25 mM each), 1ng/μL of each template (PCRs products from the two high fidelity PCRs and the mutagenic one), 3% (v/v) dimethylsulfoxide (DMSO), and 0.05 U/μL Pfu polymerase. After the last cycle

all components were added again in the PCR tube except the templates and then the second part of the assembly PCR was performed as follows: 98°C for 2 min (1 cycle), 98°C for 0.30 min, 50°C for 0.30 min, 72°C for 2 min (28 cycles) and 72°C for 10 min (1 cycle). This PCR reaction contained 0.25 µM of each primer, 1 mM dNTPs (0.25 mM each), 3% (v/v) dimethylsulfoxide (DMSO), and 0.05 U/µL Pfu polymerase. The primers used in both PCRs were RubDir sense and RubRev antisense for RubRr; 62Dir sense and 62Rev antisense for MRPro.

Genetic drift: Seven consecutive rounds of neutral genetic drift were performed on RubRr by *ep*PCR with Taq polymerase/$MnCl_2$. Once the mutant libraries were prepared according to the protocol described in the next section, aliquots of 20 µL of clones with relative activity higher than 0.7 *vs*. parental type (measured with the HTS assay described below) were transferred with the help of a liquid handler station (Freedom EVO 100, TECAN, Männedorf, Schweiz) to new 96 deep well plates containing 100 µL of LB. After 13 h of incubation at 37°C and 250 rpm and 80% relative humidity (humidity shaker Minitron-INFORS, Biogen, Spain), cultures were pooled together and plasmids were extracted with NucleoSpin Plasmid kit. This mix was then used as template to create a new generation of neutral drift. After 7 generations, 20 neutral clones were randomly selected and characterized.

Biochemical characterization

i) Production and purification. RubRr, MRPro and selected mutants were transformed into XL1-Blue and grown in 0.35 L flasks with LB-Amp at 37°C, and at a OD_{600} ~ 0.6. Rubisco production was induced with flask expression medium for 8 h at 30°C before harvesting by centrifugation (5 min at 6,000 x *g*). The cells were then frozen for 12 h at -80°C and suspended in the lysis

mixture that was described above (supplied with 1 mg/mL of lysozyme and 2 U/mL of DNase I). Additionally, they were submitted to an ultrasonic lysis by sonication and harvested by centrifugation (1 h at 12,000 x *g*). The supernatant was dialyzed in binding buffer, Tris-HCl (100 mM, pH 7.8) containing 0.5 M NaCl and 0.01 M imidazole and then passed through a Ni^{2+} Sepharose colum (HisTrap FF 5mL, GE healthcare, Sweden) attached to a fast protein liquid chromatography system (FPLC, Äkta Purifier, GE Healthcare Uppsala, Sweden). Bound protein was eluted with a gradient of elution buffer in Tris-HCl (100 mM, pH 7.8) containing 0.5 M NaCl and 0.3 M imidazole. Fractions were immediately dialyzed with successive changes to stability buffer (20mM Tris-HCl, pH 8.0, containing 50 mM NaCl) and stored at 4°C.

ii) Thermostability. The melting temperature (T_m) of purified Rubisco samples was measured in a VP-Capillary DSC (Microcal, GE Healthcare, Northhampon, USA) following a protocol described elsewhere (52). Protein solutions for calorimetric experiments were exhaustively dialyzed against Hepes buffer (25 mM, pH 8.0) containing 50 mM NaCl and the buffer from the last dialysis step was used as reference in the calorimetric experiments. Prior to scans with protein solutions, several buffer-buffer baselines were obtained to ensure proper equilibration of the calorimeter. For each protein, experiments at a scan rate of 90 K/h and a protein concentration of 10 μM were typically performed. Concentrations were determined spectrophotometrically at 280 nm using extinction coefficients and molecular weights calculated from the corresponding sequences. No significant scanning rate effects on thermal denaturation were detected when other scan rates (60 and 180 K/h) or other protein concentrations (30 μM and 0.6 μM) were employed.

iii) Expression analysis. XL1Blue cells containing parental type RubRr, MRPro, and mutants were harvested after growth and induced as indicated

above. The cells were suspended in extraction buffer (100 mM EPPS-NaOH, pH 8.0) containing 1 mM EDTA, 20 mM $MgCl_2$, 2 mM dithiothreitol, 0.043% (w/v) protease inhibitor mixture for bacterial cells and lysed with a French pressure cell (140 MPa). An aliquot of lysate sample was mixed with an equal volume of SDS buffer (125 mM Tris-HCl, pH 6.8, 4% (w/v) SDS, 20% (v/v) glycerol, 150 mM 2-mercaptoethanol, 0.01% (w/v) bromophenol blue), and the remaining was centrifuged at 38,000 x g for 15 min at 4°C. Aliquots of the supernatant were either assayed for protein content using the dye binding Pierce Coomassie Plus kit, treated with an equal volume of SDS buffer (soluble protein sample for SDS-PAGE analysis), or incubated with 25 mM $NaHCO_3$ at 25°C for 30 min before measuring Rubisco content ([2-^{14}C]carboxyarabinitol-P2 (^{14}C-CABP) binding, see below) and carboxylase activity under substrate RuBP-limiting and -saturating conditions (see below). For the specificity measurements the lysis was done as described above but without adding EDTA in the extraction buffer.

PAGE analysis. Proteins were separated by SDS-PAGE using 4–12% NuPAGE Bis-Tris Gels. The Bis-Tris gels were buffered with MES and electrophoresed at 200 V according to the supplier's instructions. Protein bands were visualized using Gelcode Blue reagent and unstained protein standard as the ladder.

Quantification of Rubisco content. Rubisco content in the XL1-Blue extracts was quantified by ^{14}C-CABP inhibitor binding after preincubation with 40 mM $NaHCO_3$ and 20 mM $MgCl_2$ as described (53). The binding assays were done in duplicate and incubated for 20 min with 15 or 45 μM ^{14}C-CABP at 25°C and the amount of Rubisco-bound ^{14}C-CABP was recovered by gel filtration and then mixed with one volume of scintillant for scintillation counting.

iv) CO_2 kinetics. K_C and K_{cat}^C was measured by $^{14}CO_2$ fixation at 25°C, pH 8.0, according to (12) in nitrogen sparged septum-capped scintillation vials. The assays were initiated by adding cell free soluble *E. coli* protein extracts (that had been preincubated for 20 min in buffer containing 20 mM $MgCl_2$ and 25 mM $NaHCO_3$) into N_2-equilibrated assay buffer EPPS-NaOH (100 mM, pH 8.0) containing 20 mM $MgCl_2$, 0.5 mM RuBP, 0.1 mg/ml carbonic anhydrase, and varying concentrations of $NaH^{14}CO_3$ (2.4-19 mM). The assays were stopped after 2 min with 0.2 volumes of 20% (v/v) formic acid and dried at 90°C, and then the residue was dissolved in 0.5 ml of water before adding 1 volume of scintillant for scintillation counting. The data was fitted to the Michaelis-Menten equation and kinetic parameters were obtained by dividing the extrapolated maximal carboxylase activity by the concentration of Rubisco active sites quantified from the ^{14}C-CABP analysis as described above.

v) RuBP kinetics. Cell free soluble *E. coli* protein extracts were preincubated with 25 mM $NaHCO_3$ for 20 min at 25 °C to activate the Rubisco. Carboxylase activities were measured using $NaH^{14}CO_3$ assays (12) containing different amounts of substrate RuBP (0–0.6 mM). Assays were buffered with EPPS-NaOH (100 mM, pH 8.0) containing 20 mM $MgCl_2$ and were performed in duplicate with unbuffered RuBP added to initiate catalysis. The assays were stopped after 70 seconds with 0.2 mL 20% (v/v) formic acid and transfer to heater block set at 90°C and dried. Then, the dried residue was dissolved in 0.5 ml of water before adding 1 volume of scintillant for scintillation counting. The K_m for RuBP was derived from fitting the data to the Michaelis-Menten equation.

vi) CO_2/O_2 specificity. $S_{C/O}$ was measured according to the method of Kane et al. (54) using purified Rubisco. Each assay (1 ml total volume) contained Rubisco and 20 µg carbonic anhydrase buffered in 30 mM triethanolamine, 15

mM MgAcetate (pH 8.1). The assays were equilibrated for 60 min with a continuous flow of humidified gas containing the desired O_2/CO_2 mole-fraction ratio produced by mixing pure CO_2 with O_2 using a series of three Wösthoff precision gas-mixing pumps. The assays were initiated by the addition of [C^{2-}[^{3}H]RuBP and after 30 minutes alkaline phosphatase added and the [^{3}H]-glycerate and [^{3}H]-glycolate recovered by anion exchange chromatography and separated by HPLC and $S_{C/O}$ calculated from the ratio of both products as described (54).

vii) DNA sequencing. Plasmid-containing mutant Rubisco genes were sequenced by GATC-Biotech. The samples were prepared with 5 µL of 100 ng/µL plasmid and 5 µL of 5 µM of each primer, Rub2 dir sense (5' TAACTTCTTGCATTATCACC 3'), Rub4 rev antisense (5' CGAAGGGCTGATTGCCCTGG 3'), Rub5 dir sense (5' AAATTAAAGAGGTATATATTAATGTATCG 3') and Rub6 dir sense (5' CGCCAAGATGCACGATTTCTAT 3') for RubRr; 62 2 dir sense (5' GCATGACTTCTACGTTCCTCCG 3'), 62 4 rev antisense (5' TTAATGAAATCGCCGCCGAGCCA 3'), 62 5 dir sense (5' CAAAAGCTGGAGCTTGCAAGCTT 3') and 62 6 dir sense (5' ATCGAGCCGGTCATGGTGCA 3') for MRPro.

viii) Protein modeling. The structural model of wild-type RubRr at a resolution of 2.6 Å (1 Å=0.1 nm) (PDB code 9RUB) was used to map the mutations and for comparison with its ancestral counterparts. MRProFir, MRPro and MRβ/γPro were modelled by the Phyre2 server (Protein Homology/analogY Recognition Engine V 2.0) (55) available at www.sbg.bio.ic.ac.uk/phyre2.
